# Improved precision in As speciation analysis with HERFD-XANES at the As *K*-edge: the case of As speciation in mine waste

**DOI:** 10.1107/S1600577522007068

**Published:** 2022-08-11

**Authors:** Emily M. Saurette, Y. Zou Frinfrock, Brent Verbuyst, David W. Blowes, Joyce M. McBeth, Carol J. Ptacek

**Affiliations:** a Department of Earth and Environmental Sciences, University of Waterloo, Waterloo, ON, Canada; b Structural Biology Center, Advanced Photon Source, Argonne National Laboratory, 9700 South Cass Avenue, Lemont, IL 60439, USA; c Department of Geology, University of Regina, Regina, SK, Canada; Bhabha Atomic Research Centre, India

**Keywords:** HERFD-XANES, geochemistry, mine waste, arsenic, linear combination fitting

## Abstract

High-energy-resolution fluorescence-detected (HERFD) X-ray absorption near-edge spectroscopy (XANES) is a spectroscopic method that allows for increased spectral feature resolution, and greater selectivity to decrease complex matrix effects compared with conventional XANES. XANES is an ideal tool for speciation of elements in solid-phase environmental samples. Accurate speciation of As in mine waste materials is important for understanding the mobility and toxicity of As in near-surface environments. In this study, linear combination fitting (LCF) was performed on synthetic spectra generated from mixtures of eight measured reference compounds for both HERFD-XANES and transmission-detected XANES to evaluate the improvement in quantitative speciation with HERFD-XANES spectra. The reference compounds arsenolite (As_2_O_3_), orpiment (As_2_S_3_), getchellite (AsSbS_3_), arsenopyrite (FeAsS), kaňkite (FeAsO­_4_·3.5H_2_O), scorodite (FeAsO_4_·2H_2_O), sodium arsenate (Na_3_AsO_4_), and realgar (As_4_S_4_) were selected for their importance in mine waste systems. Statistical methods of principal component analysis and target transformation were employed to determine whether HERFD improves identification of the components in a dataset of mixtures of reference compounds. LCF was performed on HERFD- and total fluorescence yield (TFY)-XANES spectra collected from mine waste samples. Arsenopyrite, arsenolite, orpiment, and sodium arsenate were more accurately identified in the synthetic HERFD-XANES spectra compared with the transmission-XANES spectra. In mine waste samples containing arsenopyrite and either scorodite or kaňkite, LCF with HERFD-XANES measurements resulted in fits with smaller *R*-factors than concurrently collected TFY measurements. The improved accuracy of HERFD-XANES analysis may provide enhanced delineation of As phases controlling biogeochemical reactions in mine wastes, contaminated soils, and remediation systems.

## Abstract 2

The improvement in As speciation resulting from As *K*-edge HERFD-XANES is evaluated in mine waste samples and synthetically generated mixtures of reference compounds.

## 1. Introduction

Arsenic is a metalloid that can be detrimental to human and ecological health at low concentrations. The oxidation of residual As-sulfide minerals or dissolution of As-bearing minerals in mine wastes releases dissolved As. Predicting the geochemical fate of As is important when designing remediation or prevention measures in mine wastes and other contaminated systems. Precipitation of stable and sparingly or insoluble minerals that incorporate or contain As promotes the attenuation of As. Understanding the mechanisms of As adsorption and mineralization processes, as well as the geochemical conditions that favour As attenuation, provides a foundation for the design of As remediation systems.

Arsenic mobility in environmental systems has been extensively studied (Smedley & Kinniburgh, 2002 ▸; Bissen & Frimmel, 2003 ▸). Briefly, dissolved As occurs in two principal oxidation states, As(III) or As(V), depending on the predominant geochemical conditions. The occurrence of As in mining environments has been well documented (Foster *et al.*, 1998 ▸; Morin & Calas, 2006 ▸; Walker *et al.*, 2009 ▸; Essilfie-Dughan *et al.*, 2013 ▸). Dissolved As can be attenuated by iron oxide minerals, which have a net positive surface charge under acidic to circumneutral conditions (Goldberg & Johnston, 2001 ▸; Dixit & Hering, 2003 ▸; Kanel *et al.*, 2005 ▸). Remediation efforts have also focused on removing As through reduction and the precipitation of stable Fe–As-sulfide phases (Köber *et al.*, 2005 ▸; Ludwig *et al.*, 2009 ▸; Beaulieu & Ramirez, 2013 ▸). The precipitation of these phases, notably realgar, occurs under reducing conditions and has been documented in tailings in direct contact with organic matter (Walker *et al.*, 2009 ▸; DeSisto *et al.*, 2016 ▸) and shallow aquifer sediments rich in organic matter (O’Day *et al.*, 2004 ▸). The mechanisms of As sequestration by solid phases have been studied using multiple modalities including laboratory batch and column experiments (Bertocchi *et al.*, 2006 ▸; Beesley & Marmiroli, 2011 ▸; Zhou *et al.*, 2018 ▸; Du *et al.*, 2019 ▸; Senthilkumar *et al.*, 2020 ▸; Perez *et al.*, 2021 ▸; Angai *et al.*, 2022 ▸) as well as field-scale experiments and trials (Beak & Wilkin, 2009 ▸; He *et al.*, 2010 ▸; Ludwig *et al.*, 2009 ▸; Pi *et al.*, 2017 ▸; Saunders *et al.*, 2018 ▸; Lee *et al.*, 2019 ▸).

Speciating As in solid-phase samples is challenging and can occur in three main ways: (1) wet chemical methods that rely on pre-treatments that convert the solid phase to a liquid- or gas-phase sample, *e.g.* chemical digestion and ashing (Jain & Ali, 2000 ▸); (2) destructive spectroscopic methods, such as laser ablation mass spectrometry (Resano *et al.*, 2007 ▸); and (3) non-destructive spectroscopic methods, including X-ray absorption near-edge spectroscopy (XANES) (Huggins *et al.*, 1993 ▸). XANES is a powerful method for speciating arsenic in solid samples because the amount of sample required is small, the technique is non-destructive, and the absorption edge shows a clearly measurable change with oxidation state (Foster *et al.*, 1998 ▸). A few issues that are specifically challenging in XAS analyses for environmental and geological applications are: (1) sample matrix interference (*e.g.* iron fluorescence); (2) low concentrations of elements of interest (Proux *et al.*, 2017 ▸); and (3) low proportions of geochemically important phases resulting from precipitation in microenvironments (Hochella *et al.*, 2005 ▸; Bao *et al.*, 2021 ▸). Elements of interest are often found as inclusions, substitutions, or surface-associated precipitates that are only a small fraction of the complex matrix of elements common in earth materials (Lanzirotti, 2014 ▸). Detection limits are strongly dependent on sample characteristics but in the hard X-ray regime are on the order of parts per million or parts per billion (Lanzirotti, 2014 ▸). The increased intensity of third-generation synchrotron sources, improved detectors, and other technological improvements in sensor design are important steps towards improved detection limits, but techniques to decrease the signal-to-noise ratio are critical for studying these elements at environmentally relevant concentrations (Proux *et al.*, 2017 ▸).

High-energy-resolution fluorescence detection (HERFD) was developed to improve the detection limit, increase the resolution of the features in XANES spectra (Hazemann *et al.*, 2009 ▸) and to improve site-selective EXAFS in mixed-valence compounds (Glatzel *et al.*, 2002 ▸). Many elements have been studied using this technique, including Fe, Co, Yb, As, Cu, Pb, Au, Hg and Se (Glatzel *et al.*, 2002 ▸; Safonova *et al.*, 2006 ▸; Radu *et al.*, 2007 ▸; Hazemann *et al.*, 2009 ▸; Swarbrick *et al.*, 2009 ▸; Link *et al.*, 2011 ▸; Kühn *et al.*, 2014 ▸; Proux *et al.*, 2017 ▸; Le Pape *et al.*, 2018 ▸; Gorczyca *et al.*, 2014 ▸; Nehzati *et al.*, 2021 ▸). The greater signal-to-noise ratio of HERFD (compared with conventional XAS analyses) is achieved by selecting the fluorescence signal from the element of interest using a crystal analyzer and subsequently directing the signal to a detector while the extraneous noise is lost. During routine fluorescence XANES analysis, the entire fluorescence signal is collected and a region of interest, which represents the element of interest, is selected afterward. When the energy resolution of the detected signal is greater than the core-hole lifetime [2.14 eV at the As *K*-edge (Fuggle & Inglesfield, 1992 ▸)], features that are apparent in the pre-edge, the edge and post-edge regions are better resolved (De Groot *et al.*, 2002 ▸).

This study examines reference compounds representing environmentally relevant forms of As using HERFD. Linear combination fitting (LCF) and principal component analysis (PCA) techniques were employed to evaluate improved spectral-feature resolution in HERFD-XANES compared with transmission-detected XANES for standard compounds. Arsenic speciation analyses, HERFD-XANES, and total fluorescence yield (TFY)-XANES were conducted to determine the impact of detection mode on the quantification of As species in mine waste samples. The findings of this study will allow us to evaluate the benefits of HERFD-XANES over conventional detection modes for As speciation as applied to both standard compounds and environmental samples.

## 2. Materials and methods

### 2.1. Sample collection and preparation

Arsenic reference compounds included reagent-grade arsenic trioxide (arsenolite, As_2_O­_3_) and sodium arsenate (Na_3_AsO_4_) (Sigma-Aldrich, Oakville, ON, Canada) and mineral specimens of orpiment (As_2_S_3_), getchellite (AsSbS_3_), arsenopyrite (FeAsS), kaňkite (FeAsO­_4_·3.5H_2_O), scorodite (FeAsO_4_·2H_2_O), and realgar (As_4_S_4_) (Excalibur Mineral Corp., Peekskill, NY, USA). The mineral specimens contained a combination of host rock and the mineral of interest, which was visually identified and removed from the host rock with a fine-tipped diamond Dremel to avoid contamination. Environmental samples were collected from Long Lake Mine, an abandoned gold mine in Sudbury, ON, Canada, that operated intermittently from 1909 to 1939. Samples were collected in aluminium tubing using a piston-coring technique (Starr & Ingleton, 1992 ▸). The cores were kept frozen until processing, when the cores were halved lengthwise and subsequently thawed and sampled in an anaerobic chamber (Coy Lab. Products, Inc., Grass Lake, MI, USA; 2% H_2_ and 98% N_2_ gas). Solid samples were then freeze-dried and stored at 4°C to preserve their mineralogical composition. All samples and reference compounds were finely ground with an agate mortar and pestle in an anaerobic chamber in preparation for analysis. Samples and reference compounds were transported in an anerobic canister and removed immediately prior to analysis.

### 2.2. Powder X-ray diffraction data collection and processing

Synchrotron-based powder X-ray diffraction (PXRD) was performed to confirm the identities of the arsenic reference compounds. Ground reference compounds were loaded into Kapton capillaries and sealed at both ends with Locktite 454 ep­oxy for PXRD analysis. Analyses were performed at CMCF-BM at the Canadian Light Source in Saskatoon, SK. Specifications of the beamline and endstation are fully described elsewhere (Fodje *et al.*, 2014 ▸). Samples were loaded into the sample holder and exposed to 18 keV (λ = 0.6888 Å) radiation for 10 to 30 s depending on the intensity of the observed reflections. Diffraction images were captured with a Rayonix MX300HE area detector.

Diffraction images were background-subtracted, calibrated, and integrated using *GSAS II* (Toby & Von Dreele, 2013 ▸). Diffraction images of an empty Kapton capillary and the LaB_6_ calibration standard were collected for each exposure rate and used for background subtraction and calibration. *Match!* (Version 3.10.2; Crystal Impact) was used for further data processing, including diffractogram background subtraction and phase identification with the peak search and match algorithm using the COD-Inorg REV218120 2019.09.10 database (Downs & Hall-Wallace, 2003 ▸; Gražulis *et al.*, 2009 ▸, 2015 ▸; Merkys *et al.*, 2016 ▸; Quirós *et al.*, 2018 ▸; Vaitkus *et al.*, 2021 ▸). All standard materials were positively identified (see Fig. S1 of the supporting information).

### 2.3. X-ray absorption spectroscopy (XAS) data collection

All XAS measurements were performed at 20-ID-C at the Advanced Photon Source at the Argonne National Laboratory in Lemont, IL, USA, where monochromatic X-rays in the hard X-ray regime are produced by an insertion device and a monochromator (Si 311). Ground reference compounds were spread thinly on Kapton tape and layered to achieve the appropriate thickness for collection of transmission-detected XAS. The thickness, calculated for each reference mineral using *Hephaetus* (Ravel & Newville, 2005 ▸) to achieve an absorption length between 1 and 2 to optimize the sample thickness and edge jump, ranged between 19.7 and 71.4 µm. Ground samples were pressed into a 1.524 mm-thick Teflon washer and sealed in Kapton tape for XAS analysis. Absorption lengths calculated for the reference materials were assumed to be appropriate for the mine waste samples. Ion chambers were used to measure the incident and transmitted X-ray intensity. The beam spot-size on the sample was 42 µm × 30 µm (w × h). A Vortex four-element Si drift detector was used to collect the TFY spectra and was positioned at 45° from the sample surface (90° from the incident beam). The energy resolution of the TFY detector is 100 eV or greater. The high-energy-resolution signal was measured by a Pilatus 100K area detector after being focused with a bent Si (911) crystal analyzer, at a Bragg angle of 80.5°, in Rowland geometry with a radius of 0.5 m (Fig. S2). The energy resolution of the crystal analyzer is approximately 1 eV. Normally, filters can be applied upstream of the sample to attenuate and lower the intensity of the incident beam to optimize the count rate for fluorescence yield (FY) detection; however, retaining the maximum signal intensity is critical for HERFD. When FY and HERFD were measured simultaneously, the FY count rate was optimized using aluminium foil to directly filter the signal before it entered the detector.

### 2.4. XAS data processing

All XAS data were pre-processed using *LARCH* (Newville, 2013 ▸). Normalization and post-edge flattening were applied to all XANES spectra. The signal-to-noise ratio for each reference and sample spectra was calculated by processing all available independent scans and estimating the statistical error using the pre-edge and EXAFS region (Sayers, 2000 ▸). The XANES region was not included in the statistical error or signal-to-noise calculation to avoid biasing the results with variations in the spectra caused by beam damage. LCF was used to determine the percentage of As associated with each species. Spectra were collected for reference compounds arsenolite (As_2_O_3_), orpiment (As_2_S_3_), getchellite (AsSbS_3_), arsenopyrite (FeAsS), kaňkite (FeAsO_4_·3.5H_2_O), scorodite (FeAsO_4_·2H_2_O), sodium arsenate (Na_3_AsO_4_), and realgar (As_4_S_4_). Computer-generated spectra of mixtures were created with the spectra from each of the reference compounds, measured in both HERFD and transmission. All computer-generated mixtures were a combination of four reference compounds, each with a minimum contribution of 5% of the total mixture; these limitations were selected to represent speciation with a commonly acceptable number of fit components and detection limit. The computer-generated mixtures were analyzed with LCF and PCA. The datasets of mixtures were created with both HERFD and transmission reference compounds and each unique reference compound was included in 3500 synthetic spectra. A dataset was generated for each of the 70 combinations of four reference compounds to decrease sampling bias. A set of 100 random mixture ratios was generated using the *NumPy* module (Van Der Walt *et al.*, 2011 ▸; Oliphant, 2006 ▸) and stored in a *Pandas DataFrame* (McKinney, 2010 ▸). The set of 100 random mixture ratios was constant for each combination of reference compounds. The reference compounds were then added in the specified ratios with the addition of random noise from a normal distribution equal to 2.5% of the edge jump to create the synthetic spectra for LCF and PCA. Random noise was added to simulate the reduction of the signal-to-noise ratio that often occurs in natural samples.

LCF was applied using a non-negative least-squares algorithm (Lawson & Hanson, 1987 ▸) to fit the synthetic spectra with the reference compounds. The non-negative least-squares algorithm applies the boundary condition that no standard can have a coefficient less than zero. LCF was completed for each synthetic spectrum in all random-mixture datasets using an in-house code (see supporting information). LCF analysis on the mine waste samples was completed in *Athena* (Ravel & Newville, 2005 ▸) and all combinations of standards were evaluated using the Hamilton test as described by Downward *et al.* (2007 ▸). When multiple fits were not significantly better than the fit with the lowest *R* ^2^ value, the best fit was chosen from the statistically equivalent fits based on supporting observations, including optical mineralogy, scanning electron microscopy and energy-dispersive spectroscopy, and PXRD (Verbuyst, 2020 ▸).

PCA was used to determine the six principal components with the highest associated explained variance from the dataset of synthetic spectra derived from the arsenopyrite, arsenolite, sodium arsenate, and scorodite reference compounds. These four references were chosen for their visually distinct spectra. Linear dimensionality reduction with PCA was performed on the dataset with the singular value decomposition algorithm from the *scikit-learn* module (Pedregosa *et al.*, 2011 ▸), and the explained variance for each component was determined. Target transformation was applied to fit the experimentally measured reference compounds that were used to create the databases with the mathematical components derived from the PCA. The target transformation was completed using the non-negative least-squares algorithm (Lawson & Hanson, 1987 ▸) implemented in the *SciPy* library (Virtanen *et al.*, 2020 ▸), with one, two, three, and four components to evaluate the quality of fit as the explained variance increased. The residual was calculated as the difference between the standard and the target transformation for each number of components and the standard deviation of the residual from *y* = 0 was calculated.

## 3. Results and discussion

### 3.1. Arsenic sulfides

**Figure 1:**
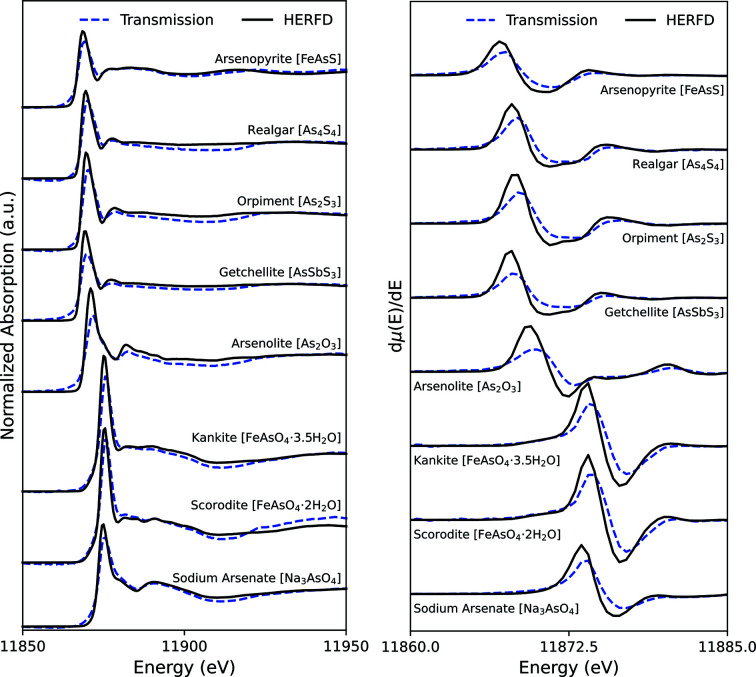
A comparison of normalized As *K*-edge XANES spectra collected in HERFD and transmission-detection modes for arsenic compounds associated with mine waste (left panel) and the first derivatives (right panel).

Primary arsenic sulfides typically occur in association with hydro­thermal ore bodies targeted for precious and base metal mining (Bondu *et al.*, 2020 ▸), whereas secondary arsenic sulfides have been observed to occur where reducing environments coincide with sufficient aqueous As and S concentrations (O’Day *et al.*, 2004 ▸; Walker *et al.*, 2009 ▸; DeSisto *et al.*, 2016 ▸; Galloway *et al.*, 2018 ▸). The As-sulfide species arsenopyrite, realgar, orpiment, and getchellite all have similar post-white-line features in the XANES region (Fig. 1 ▸). The white-line position for arsenopyrite in HERFD is 11868.0 eV and approximately 1 eV lower in energy than the other As-sulfides, indicative of the −I As oxidation state in the arsenopyrite structure (Table 1 ▸). The post-white-line structure for arsenopyrite features a broad oscillation containing two small local maxima at 11876.5 and 11882.0 eV (Table 1 ▸). As-sulfides without Fe (realgar, orpiment, and getchellite) resulted in nearly identical spectra (Fig. 1 ▸), likely because of their similar compositions and bonding environments with respect to As. Theoretical spectra from density functional theory (DFT) calculations for realgar and orpiment show good agreement with experimental data and the density of states elucidate the contributions of the *p*-orbital mixing between As and neighbouring S atoms (Le Pape *et al.*, 2018 ▸). HERFD measurements on standard mineral samples showed improvement in the sharpness of the absorption edge. A pre-edge structure is not present in the spectra for any of the compounds, but a post-white-line structure is observed for most compounds (Fig. 1 ▸).

### 3.2. Arsenic oxides

As-oxides are often formed in mine waste as secondary precipitates (Murciego *et al.*, 2019 ▸). The As-oxides analyzed in this study contain As(III) (arsenolite) or As(V) (kaňkite, scorodite, and sodium arsenate) oxidation states (Table 1 ▸). The arsenolite white line is observed at 11870.5 eV in HERFD and is approximately 5 eV lower than for the As(V)-oxide compounds (Table 1 ▸), which is consistent with previous observations of the effect of oxidation state on the white-line energy (Filimonova *et al.*, 2020 ▸). The As—O bond length is similar for all of the As(V) compounds at approximately 1.67 Å, whereas the As—O bond length is longer for the As(III) containing arsenolite because of the lack of As=O double bond and higher co-ordination number of 6 compared with As(V) with a co-ordination number of 4. All measured As-oxide species have a more visible post-white-line structure in the HERFD-XANES spectra compared with the transmission-detected XANES spectra (Fig. 1 ▸).

### 3.3. Comparison of HERFD and transmission XAS measurements

The resolution increases observed in the HERFD-XANES spectra are similar to other studies (Proux *et al.*, 2017 ▸; Le Pape *et al.*, 2018 ▸). The signal-to-noise ratios of HERFD-XANES spectra were 1.5–4.0 times greater than the signal-to-noise ratios of transmission-detected spectra (Fig. S6, Table S1). A small shift of −0.5 to −1 eV is observed in white-line energy in HERFD measurements compared with transmission measurements (Table 1 ▸), which is attributed to the emission energy selected for the experiment (Le Pape *et al.*, 2018 ▸). HERFD-XANES does not measure the full absorption cross-section, unlike transmission or TFY detection, because it does not collect the full fluorescence signal from the As relaxing fluorescence. HERFD-XANES could miss information that may be a part of the fluorescence signal and is evident in a resonant inelastic X-ray scattering (RIXS) profile. The capacity for highly selective detection is the basis for site-selective HERFD-XANES. Portions of the fluorescence signal originate from different molecular orbital properties, such as the spin states of iron compounds (Glatzel *et al.*, 2002 ▸). Le Pape *et al.* (2018 ▸) demonstrated that HERFD critically requires the selection of a consistent emission energy for all reference compounds and samples if the samples contain an unknown mixture of standard compounds to ensure sample spectra can be compared. In contrast, transmission and partial FY-detected XAS experiments capture the complete absorption cross-section and emission energy and are not subject to the same concern. The HERFD method used in this study established a consistent detection signal by using a stationary crystal analyzer and a non-energy-selective area detector to detect signal intensity (Fig. S2); variations in set-up angles were minimized by the magnetically positioned sample holder and consistent sample mounting technique ensuring the sample surface position was unchanged between samples and references. This configuration allows comparison of measurements across samples and reference compounds.

**Figure 2:**
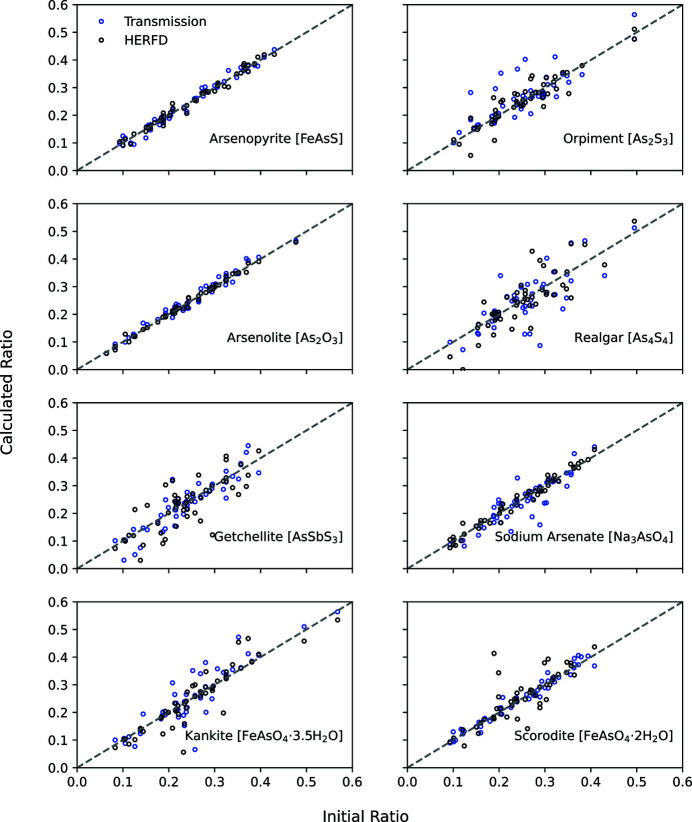
A comparison of the initially generated ratio that was used to create the synthetic standard and the ratio determined from LCF. HERFD results are in black and transmission results are in blue. LCF that perfectly replicated the data would result in points that lie along unity (grey dashed line). Fifty of the total 3500 results were randomly selected for visualization to improve the readability of the figure.

The standard error of regression was lower for the HERFD datasets for synthetically generated mixtures of arsenopyrite, orpiment, arsenolite, and sodium arsenate XAS spectra relative to transmission-detected XAS analyses (Fig. 2 ▸, Table 2 ▸). The standard error of regression either increased or remained the same for realgar, getchellite, scorodite and kaňkite (Fig. 2 ▸, Table 2 ▸). The absolute difference in the standard error of regression between HERFD and transmission-detected XAS ranged from 0.2 to 2.5% (Table 2 ▸), therefore any improvement in LCF is likely to be small. Sharper absorption edges may improve the accuracy of determining the oxidation state of arsenic, but the measured fluorescence must be carefully selected during HERFD measurements as the measurements do not represent the full information content of the absorption cross-section (Glatzel & Juhin, 2014 ▸). LCF for synthetically generated mixtures demonstrates the benefits in HERFD detection for an ideal sample where the concentration of As is not prohibitively low and the matrix does not interfere with the measured fluorescence signal.

**Figure 3:**
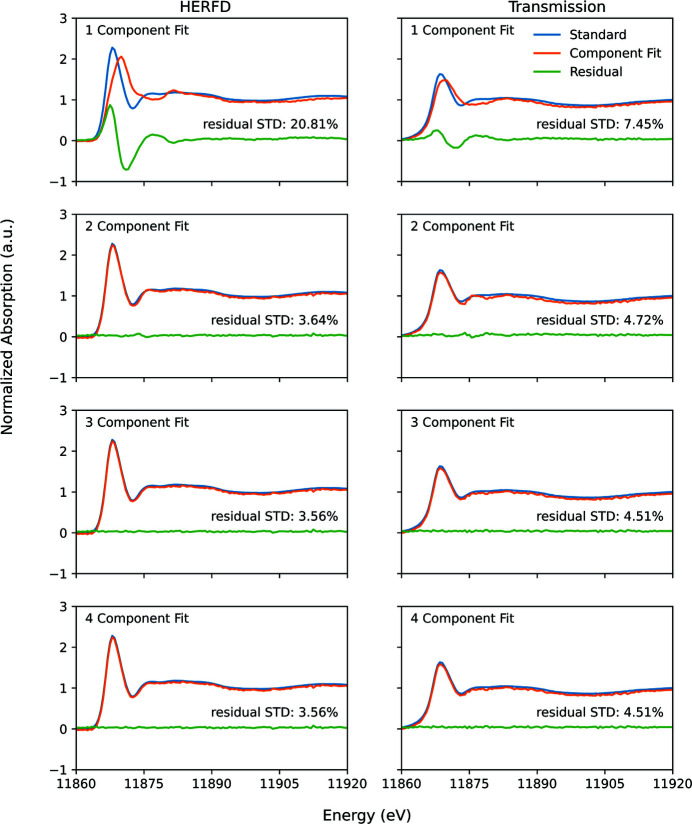
Demonstration of target transformations for HERFD and transmission spectra for the arsenopyrite standard.

**Figure 4:**
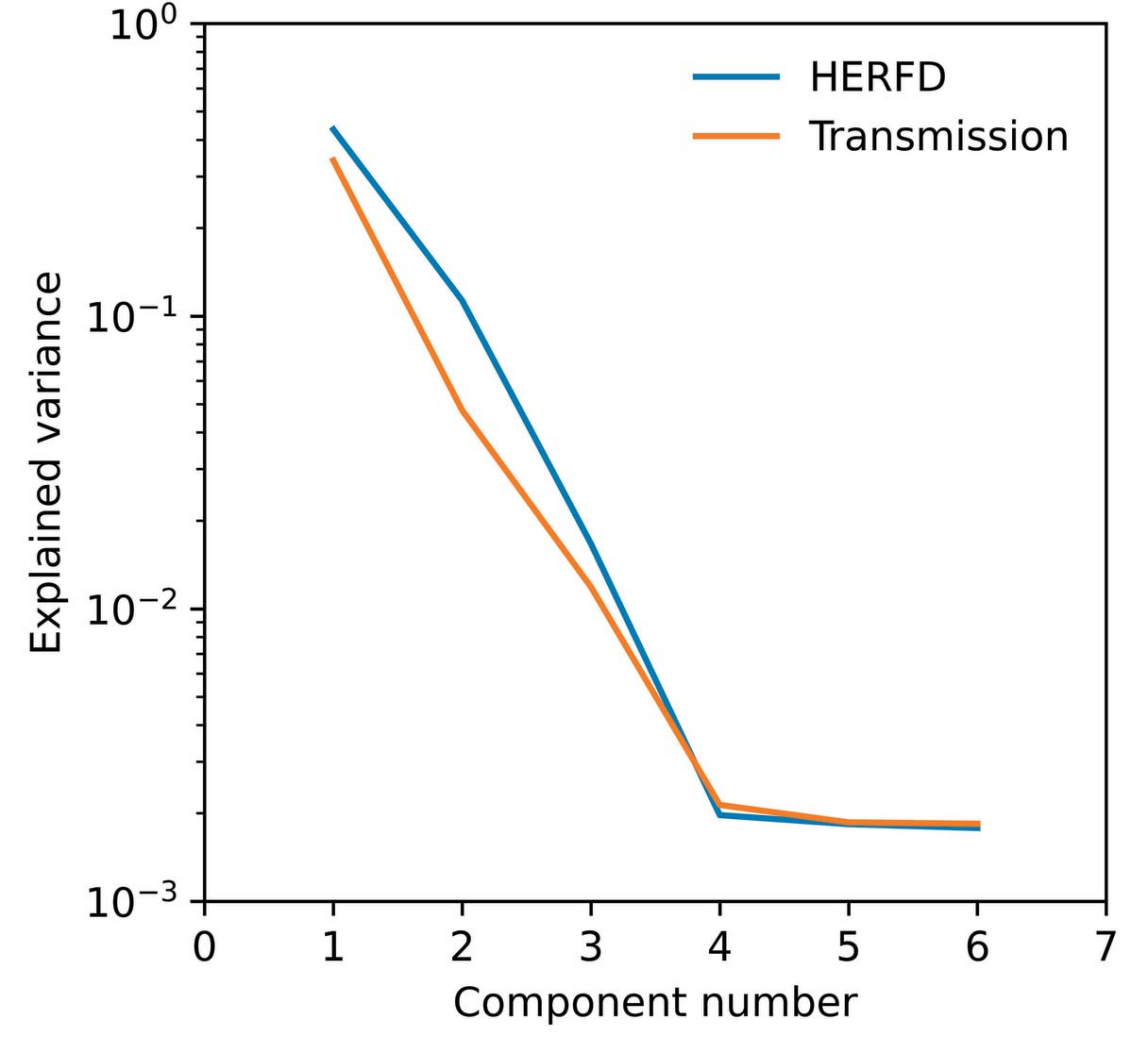
Scree plot for PCA components and the associated explained variance for both HERFD and transmission datasets of synthetically generated mixtures of various ratios of arsenopyrite, arsenolite, sodium arsenate, and scorodite. Note that, with HERFD, each of the first three PCA components can explain a greater amount of the variance in the dataset.

PCA of the dataset of computer-generated mixtures of arsenopyrite, arsenolite, sodium arsenate, and scorodite and subsequent target transformation demonstrate that three principal components can be used to satisfactorily fit the arsenopyrite (Fig. 3 ▸), arsenolite (Fig. S3), scorodite (Fig. S4), and sodium arsenate (Fig. S5) reference spectra for both the HERFD and transmission data, but additional components did not further decrease the residual standard deviation. The synthetic mixtures of arsenopyrite, arsenolite, sodium arsenate, and scorodite were selected for PCA and target transformation because each had unique spectral features (Fig. 1 ▸). Manceau *et al.* (2014 ▸) found that using the eigenvalues of the principal components could result in underestimation of the number of real components. In a study performed on datasets of HERFD- and TFY-XANES synthetic mixtures of Hg compounds, the authors concluded that PCA of HERFD-XANES datasets more accurately identified the real number of components than PCA on the fluorescence data (Proux *et al.*, 2017 ▸). Similar results are found in the present study; the respective components for HERFD have larger eigenvalues in the covariance matrix than the equivalent components derived from the transmission dataset (Fig. 4 ▸).

Overall, PCA is beneficial as a tool to estimate the number of real components; however, it is critical to interpret PCA with caution. PCA cannot definitively determine the number of real components because the noise level of the dataset and number of spectra that are included in the dataset used to generate the PCA affect these coefficients. In addition, the true values of real components in the system can be underestimated if two or more components are statistically related (Manceau *et al.*, 2014 ▸). The ability of statistical techniques such as PCA to differentiate the contribution of species that do not have unique spectral features is weaker than when unique spectral features are present (Levina *et al.*, 2007 ▸).

### 3.4. Application to field samples

**Figure 5:**
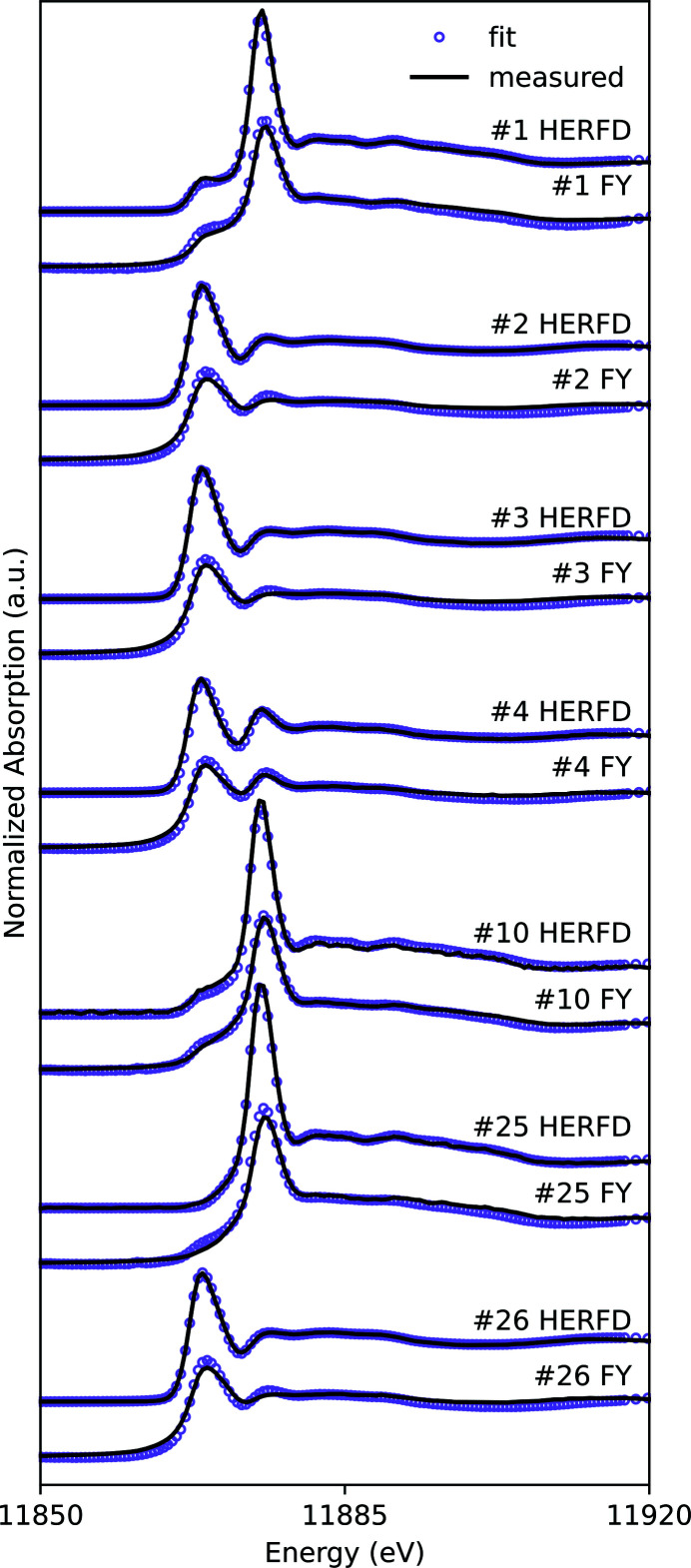
A comparison between the fits obtained with LCF for seven As-bearing tailings samples for both HERFD and FY detection.

Signal-to-noise ratios did not improve for field samples with HERFD and the mean improvement factor was 0.81 (*n* = 30, SD = 0.39, Table S1). The longer air-path to the area detector used for HERFD measurements could attenuate up to 56% of the As *K*α fluorescence emission which would decrease the signal-to-noise ratio. A helium bag placed in the air-path would decrease the attenuation and improve the signal-to-noise ratio. However, improved speciation using HERFD-XANES was observed in samples of As-bearing mill tailings collected from the Long Lake mine, which were identified to have a binary mixture of arsenopyrite and scorodite or kankite (Fig. 5 ▸), representing 7 of the 30 field samples analyzed. PCA analysis on the 7 field samples and target transformation performed on the HERFD and transmission reference spectra confirmed that the first two principal components explain most of the variance (Fig. S7) and arsenopyrite, scorodite and kankite are likely chemical components of the dataset (Figs. S8, S9, S10) whereas getchellite, orpiment, realgar, arsenolite and sodium arsenate are unlikely to be chemical components of the dataset (Figs. S11, S12, S13, S14, S15). On average, the LCF on HERFD-XANES spectra obtained from Long Lake samples with binary mixtures resulted in fits with significantly lower *R* ^2^ (*n* = 7, *P* = 0.03) than fits on FY XANES spectra obtained from the same samples (Table 3 ▸). The quantity of As identified as arsenopyrite and scorodite in the same sample varied by 2–15% between LCF performed on HERFD-XANES spectra and LCF performed on the TFY-XANES spectra (Table 3 ▸). Neither arsenopyrite nor scorodite were consistently under- or overestimated in LCF performed on either detection method (Table 3 ▸). The improved fits for HERFD-XANES spectra may be attributed to a combination of the improved spectral resolution and the ease of comparison between standard and sample spectra.

Sample spectra collected using FY detection were fit with reference compounds measured in transmission detection, which could be a source of misfit between the sample and standard spectra. Spectra collected in HERFD or TFY from thick samples containing high As concentrations (Table S2) could be impacted by over-absorption (self-absorption) that dampens the amplitude of the XAFS oscillations (Newville, 2014 ▸). Over-absorption is a result of the fluorescence emission from the sample, and can be present in HERFD and conventional TFY measurements (Nehzati *et al.*, 2021 ▸). Over-absorption corrections could not be made because As concentrations (Table S2) were not measured on the same samples used for XAS measurements. If over-absorption is present in the samples, the quality of the fits in both HERFD and TFY would be impacted.

Field samples that had best fits containing As(−I) (arsenopyrite), As(III)–S (getchellite), As(III)–O (arsenolite) and As(V) (sodium arsenate) (Table S3) showed no improvement in *R* ^2^ (*n* = 23, *P* = 0.25) with HERFD-XANES spectra. The lack of improved fit quality may be attributed to reference materials that do not exactly represent, but have similar molecular bonding environments to, the species present in the sample. Getchellite, orpiment and realgar are rare As-sulfide minerals that are not often identified as secondary minerals in oxidized mine waste environments; however, the spectra for these minerals are similar to arsenical pyrite (Le Pape *et al.*, 2018 ▸) which may form in oxidizing shallow groundwater systems (Deditius *et al.*, 2008 ▸; Le Pape *et al.*, 2017 ▸). Arsenolite and sodium arsenate have similar molecular bonding environments to As(III) and As(V) bound to mineral surfaces and similar spectra (Beak & Wilkin, 2009 ▸). The misfit caused by using proxy references may obscure the improvement in LCF and reiterates the need for appropriate references.

The spectral resolution of U- (Kvashnina *et al.*, 2013 ▸; Leinders *et al.*, 2017 ▸; Le Pape *et al.*, 2020 ▸), Hg- (Proux *et al.*, 2017 ▸), Se- (Bissardon *et al.*, 2019 ▸; Nehzati *et al.*, 2021 ▸), As- (Le Pape *et al.*, 2018 ▸), Au- (Tagirov *et al.*, 2016 ▸; Filimonova *et al.*, 2020 ▸), and Cu-XANES (Tagirov *et al.*, 2016 ▸) in geologically or biologically relevant phases have shown notable improvement with HERFD. The high-resolution spectra improve speciation and enable the more accurate determination of oxidation state in samples with low concentrations and/or complex matrices (Proux *et al.*, 2017 ▸). Samples of As-bearing tailings at Long Lake have complex matrices, containing between 17600 and 68100 p.p.m. of Fe, whereas As concentrations are between 1039 and 43700 p.p.m. (Table S2). Arsenopyrite was identified in some of the tailings samples with conventional XRD measurements, and Fe–As–O-containing phases were previously identified with SEM-EDS (Verbuyst, 2020 ▸). This study demonstrates that HERFD for As *K*-edge XANES results in a significant refinement in LCF for samples containing a binary mixture of arsenopyrite and scorodite or kankite in this concentration range with complex Fe-rich matrices. The As and Fe concentrations in Long Lake tailings samples are representative of concentrations present in mine waste solids at sites across Canada where As is a contaminant of interest (Fawcett *et al.*, 2015 ▸; DeSisto *et al.*, 2016 ▸). Mine waste solids and solids derived from remediation systems such as permeable reactive barriers can have concentrations in the p.p.m. to p.p.b. range (Beak & Wilkin, 2009 ▸); determination of the speciation of As in these samples may also be improved with HERFD-XANES.

Reference compounds that would greatly improve the ability to perform LCF on samples derived from As-treatment experiments include As(III) and As(V) sorbed to various relevant iron oxides and iron hy­droxy-sulfates, such as goethite, ferrihydrite, jarosite, and hematite. XANES and EXAFS studies show that As sorbs to and is incorporated into secondary precipitates (Beak & Wilkin, 2009 ▸; Parviainen *et al.*, 2012 ▸). HERFD may not provide sufficient resolution to differentiate As bound to these phases, but HERFD may provide sufficient information to more accurately differentiate sorbed As from As retained in discrete arsenic oxide or arsenic sulfide precipitates.

## 4. Conclusions

HERFD-XANES provides resolution of unique spectral features that may not be detectable with transmission-detection XAS analyses, and this additional resolution enhances LCF fitting when the appropriate standards are available. Slight improvements in spectral resolution are also expected when As is dilute or in a complex matrix, which is one of the main benefits of HERFD for environmental samples. Developing a database of environmentally relevant As reference compounds is vital to the application of LCF with HERFD-XANES to quantitatively determine the speciation of As in mine wastes and other haza­rdous materials. Use of standard spectra from a HERFD-XANES database is critical with respect to the application of this technique (*e.g.* use of the spectra provided in this manuscript for PCA and LCF analysis of sample data with use the same beamline geometry to ensure the data are comparable).

## Supplementary Material

Details on Datasets 1 and 2. Figures S1 to S15. Tables S1 to S3. DOI: 10.1107/S1600577522007068/ye5018sup1.pdf

## Figures and Tables

**Table 1:** Molecular and crystallographic information for the As reference compounds and locations of identified peaks in the HERFD-XANES and transmission-detected XANES spectra

Compound	As oxidation state	Crystal system	As—Nearest-Neighbour bond length	Peak 1 (eV)	Peak 2 (eV)	Peak 3 (eV)	Reference
				HERFD; transmission			
Arsenopyrite (FeAsS)	−I	Monoclinic	As—S 2.346 Å	11868.0;	11876.5;	11882.0;	Fuess *et al.* (1987 ▸)
		*P*2_1_/*d*	As—Fe 2.336–2.375 Å	11868.5	11878.0	11883.5	
Realgar (As_4_S_4_)	II	Monoclinic	As—As 2.570–2.571 Å	11869.5;	11877.5;	11884.0;	Kyono *et al.* (2005 ▸)
		*P*2_1_/*c*	S—S 2.230–2.249 Å	11870.0	11877.5	–	
Orpiment (As_2_S_3_)	III	Monoclinic	As—S 2.283 (5) Å	11869.5;	11878.5;	–;	Mullen & Nowacki (1972 ▸)
		*P*2_1_/*c*		11870.5	11879.0	–	
Getchellite (AsSbS_3_)	III	Monoclinic	(As,Sb)—S 2.287–2.460 Å	11869.5;	11877.5;	–;	Kyono & Kimata (2004 ▸)
		*P*2_1_/*a*		11870.0	11877.5	–	
Arsenolite (As_2_O_3_)	III	Cubic, isometric	As—O 1.786 (2) Å	11871.0;	11882.0;	–;	Ballirano & Maras (2014 ▸)
		*Fd*3*m*		11871.5	11882.5	–	
Kaňkite (FeAsO_4_·3.5 H_2_O)	V	Monoclinic	–	11875.0;	11882.5;	11890.0;	Cech *et al.* (1976 ▸)
		*P*2		11875.5	–	11890.0	
Scorodite (FeAsO_4_·2H_2_O)	V	Orthorhombic	As—O 1.670 (5)–1.685 (4) Å	11875.5;	11881.5;	11891.0;	Kitahama *et al.* (1975 ▸)
		*Pbca*		11876.0	11882.0	11891.0	
Sodium arsenate (Na_3_AsO_4_)	V	Monoclinic	As—O 1.661 (4)–1.679 (4) Å	11875.0;	11890.5;	–;	Ferraris & Chiari (1970 ▸)
		*P*2_1_/*n*		11875.5	11891.0	–	

**Table 2:** Standard error of regression for LCF results for each reference compound in all mixture combinations in both transmission-detected XANES and HERFD-XANES A lower standard error of regression indicates LCF results that more closely agree with the true mixture ratios.

	Standard error of regression (%)							
Detection method	Arsenopyrite	Arsenolite	Getchellite	Kaňkite	Orpiment	Realgar	Sodium arsenate	Scorodite
Transmission	1.8	1.4	3.2	3.9	4.6	4.6	3.4	1.7
HERFD	1.4	0.9	5.6	4.1	4.1	7.1	1.4	4.1

**Table 3:** LCF results for spectra from Long Lake mine tailings samples with a binary mixture of arsenopyrite and scorodite or kankite Spectra were collected simultaneously with both HERFD and TFY detection. As(−I) is represented by arsenopyrite and As(V) is either scorodite or kankite. The LCF results for all spectra are included in Table S3 of the supporting information.

Sample #	Detection Mode	As(−I) (% As atoms)	As(V) (% As atoms)	*R* ^2^
1	HERFD	20 ± 1	80 ± 1	0.0036
1	TFY	29 ± 1	71 ± 1	0.0081
2	HERFD	96 ± 1	4 ± 1	0.0002
2	TFY	92 ± 2	8 ± 1	0.0128
3	HERFD	98 ± 1	2 ± 1	0.0004
3	TFY	94 ± 1	6 ± 1	0.0085
4	HERFD	87 ± 1	13 ± 1	0.0046
4	TFY	85 ± 1	15 ± 1	0.0081
10	HERFD	13 ± 1	87 ± 1	0.0065
10	TFY	21 ± 1	79 ± 1	0.0031
25	HERFD	–	100 ± 1	0.0071
25	TFY	15 ± 2	85 ± 2	0.0125
26	HERFD	97 ± 1	3 ± 1	0.0003
26	TFY	93 ± 2	7 ± 1	0.0114
