# Improved precision in As speciation analysis with HERFD-XANES at the As *K*-edge: the case of As speciation in mine waste. Corrigendum

**DOI:** 10.1107/S1600577523006069

**Published:** 2023-10-18

**Authors:** Emily M. Saurette, Y. Zou Finfrock, Brent Verbuyst, David W. Blowes, Joyce M. McBeth, Carol J. Ptacek

**Affiliations:** a Department of Earth and Environmental Sciences, University of Waterloo, Waterloo, ON, Canada; b Structural Biology Center, Advanced Photon Source, Argonne National Laboratory, 9700 South Cass Avenue, Lemont, IL 60439, USA; c Department of Geology, University of Regina, Regina, SK, Canada; Bhabha Atomic Research Centre, India

**Keywords:** HERFD-XANES, geochemistry, mine waste, arsenic, linear combination fitting

## Abstract

The name of an author in the article by Saurette *et al.* (2022) [*J. Synchrotron Rad.* **29**, 1198–1208] is corrected.

## Abstract 2

Corrigendum to the article by Saurette *et al.* (2022) [*J. Synchrotron Rad.* **29**, 1198–1208].

The name of the second author in the article by Saurette *et al.* (2022 ▸) is incorrectly given as Y. Zou Frinfrock. The correct spelling is Y. Zou Finfrock, as given above.
