# Supplementary material for: Improved precision in As speciation analysis with HERFD-XANES at the As K-edge: the case of As speciation in mine waste
Source: J Synchrotron Radiat. 2022 Aug 11;29(Pt 5):1198–208. doi: 10.1107/S1600577522007068 (PMC9455218; doi:10.1107/S1600577522007068)
Supplement: Supplementary file 1 [file s-29-01198-sup1.pdf]

JOURNAL OF SYNCHROTRON RADIATION

Volume 29 (2022)

Supporting information for article:

Improved precision in As speciation analysis with HERFD-XANES at the As K-edge: The case of As speciation in mine waste

Emily M. Saurette, Y. Zou Frinfrock, Brent Verbuyst, David W. Blowes, Joyce M. McBeth and Carol J. Ptacek

Dataset 1:

This dataset includes raw XAS data collected in HERFD and transmission-detection mode for As-bearing reference minerals. This data was collected at 20-ID-C at the Advanced Photon Source at the Argonne National Laboratory. The dataset can be accessed at: https://doi.org/10.5281/zenodo.5669967.

Dataset 2:

This dataset includes raw powder X-ray diffraction data for arsenic-bearing reference minerals, an empty Kapton™ capillary for background subtraction, and, LaB6 for the calibration of instrument parameters. This data was collected at the Canadian Macromolecular Crystallography Facility, beamline 08B1-1, at the Canadian Light Source. The dataset can be accessed at: https://doi.org/10.5281/zenodo.5670309.

Data processing code:

The data was partially processed using code that can be accessed at: https://doi.org/10.5281/zenodo.6394767.

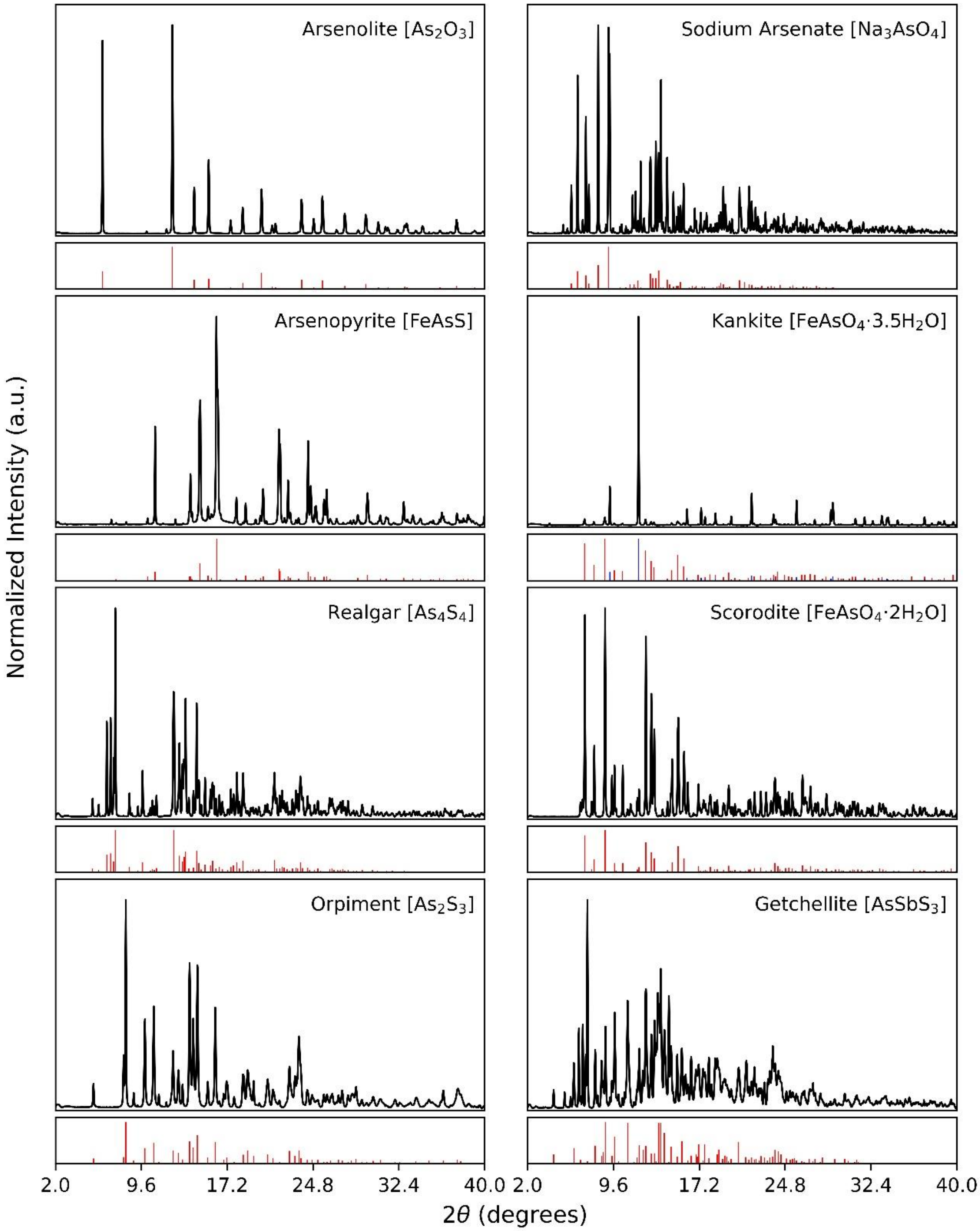

Figure S1. PXRD patterns for the As-bearing reference minerals. Normalized peak positions and intensities from the COD database are included below each measured pattern. Note that the measured kankite PXRD pattern includes quartz (shown in blue) that originated from the host rock it was sampled from.

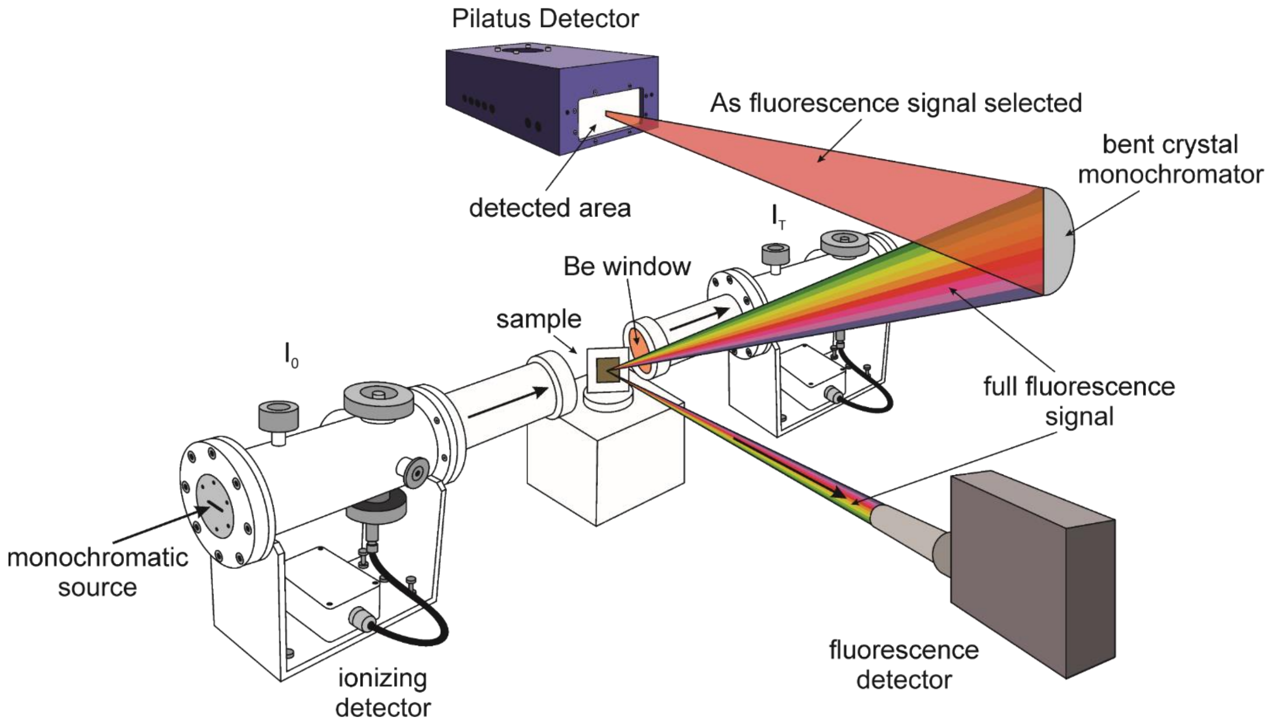

Figure S2. A schematic diagram of the experimental set up.

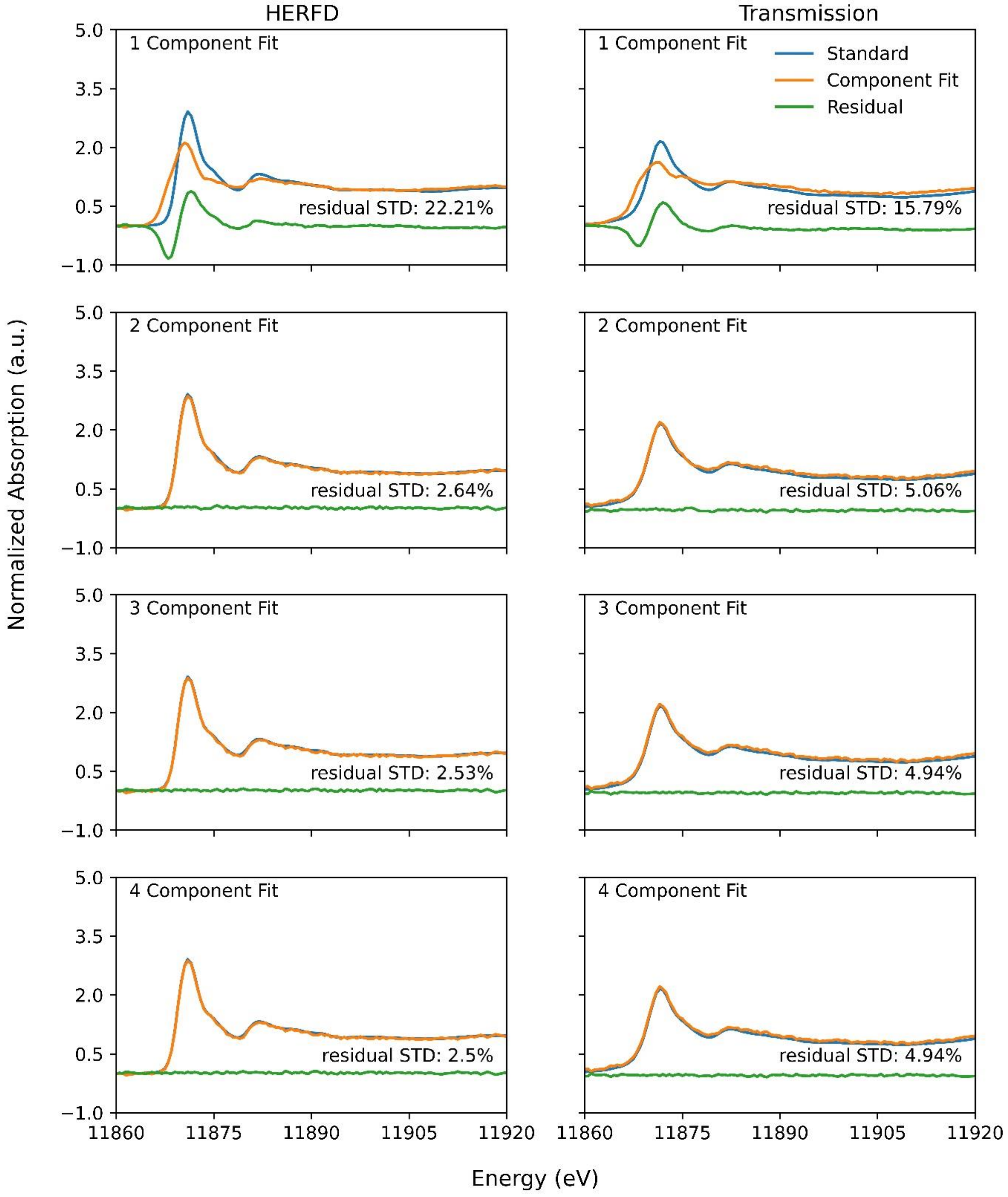

Figure S3. Demonstration of target transformations for HERFD and transmission spectra for the arsenolite standard.

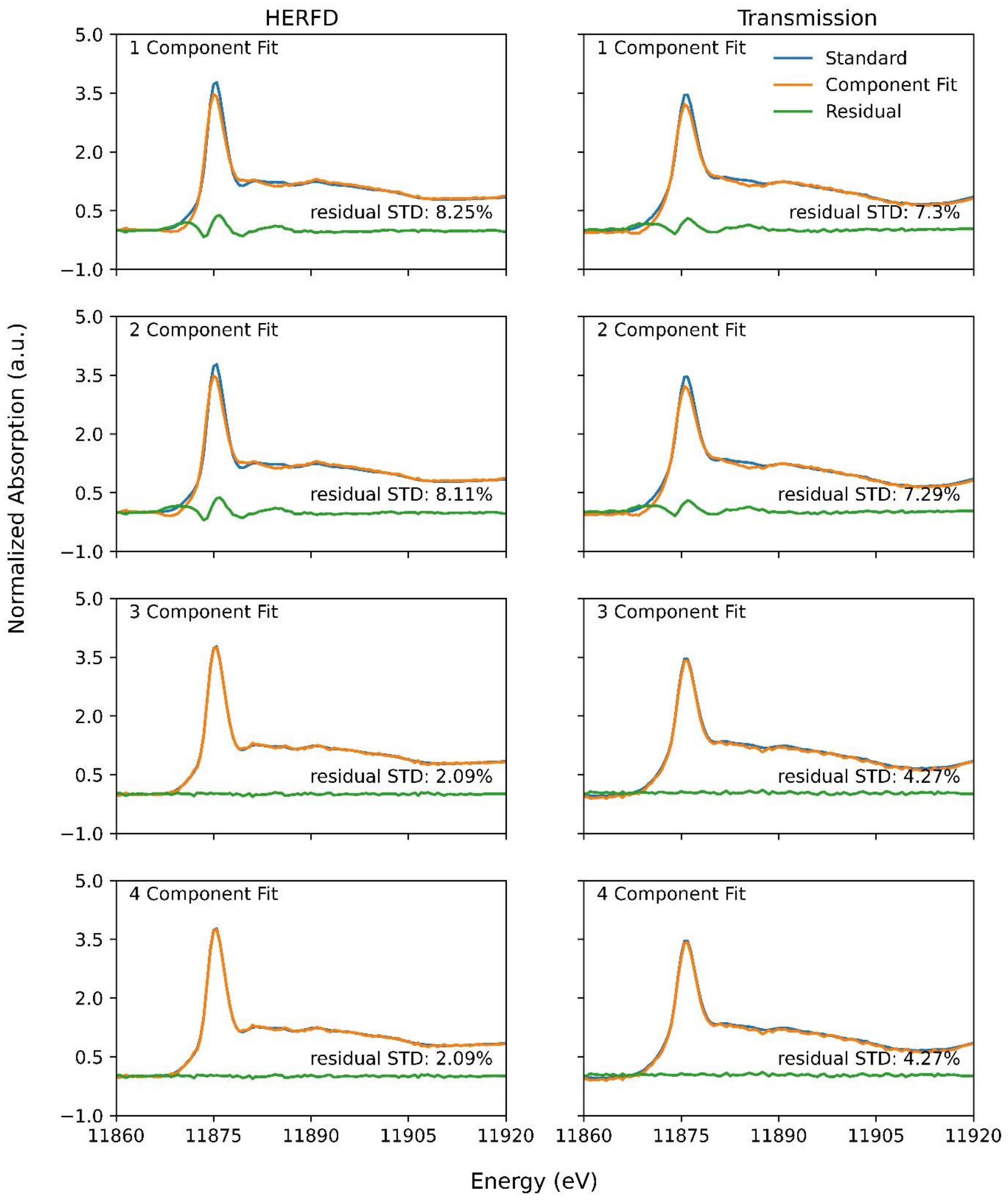

Figure S4. Demonstration of target transformations for HERFD and transmission spectra for the scorodite standard.

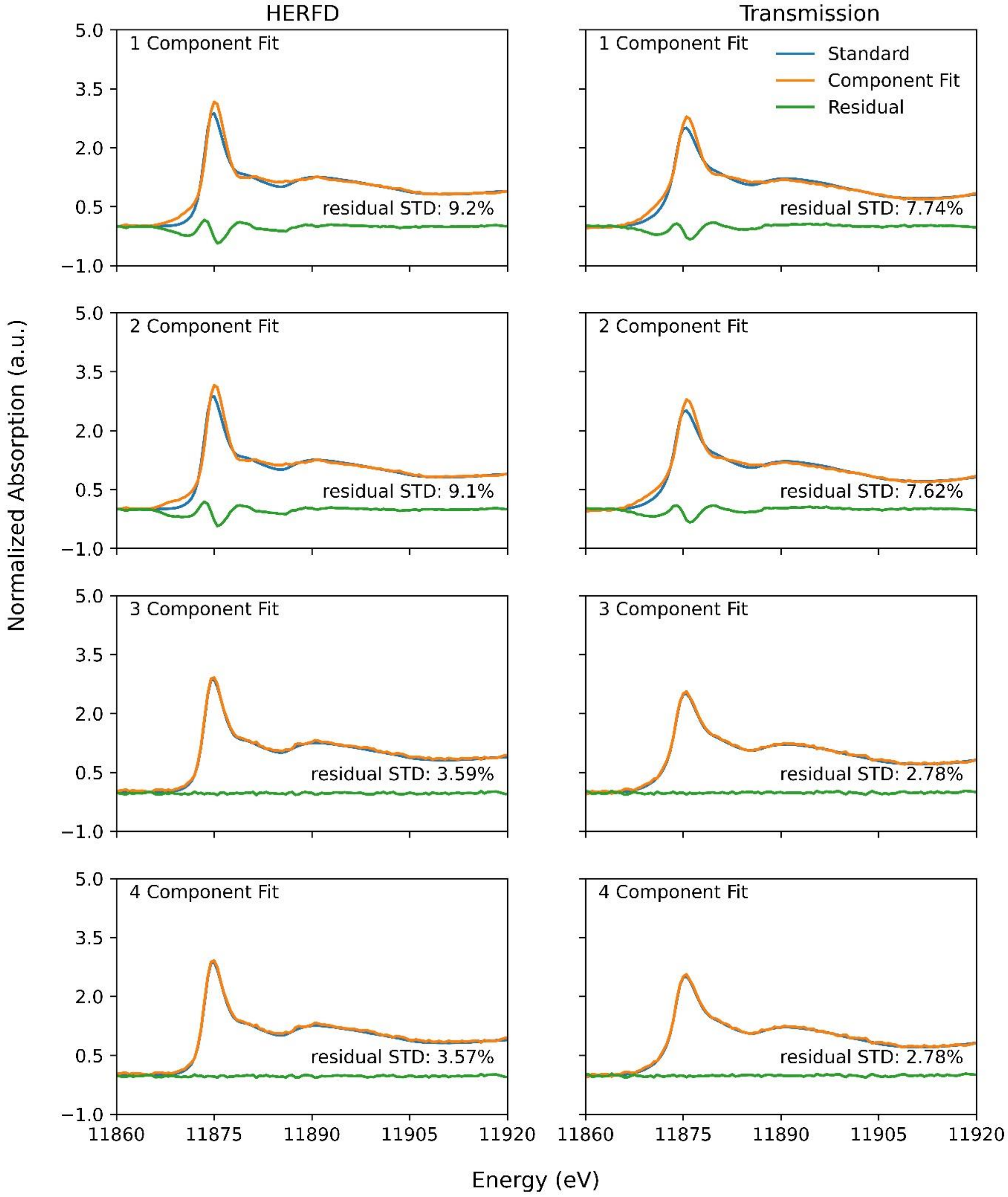

Figure S5. Demonstration of target transformations for HERFD and transmission spectra for the sodium arsenate standard.

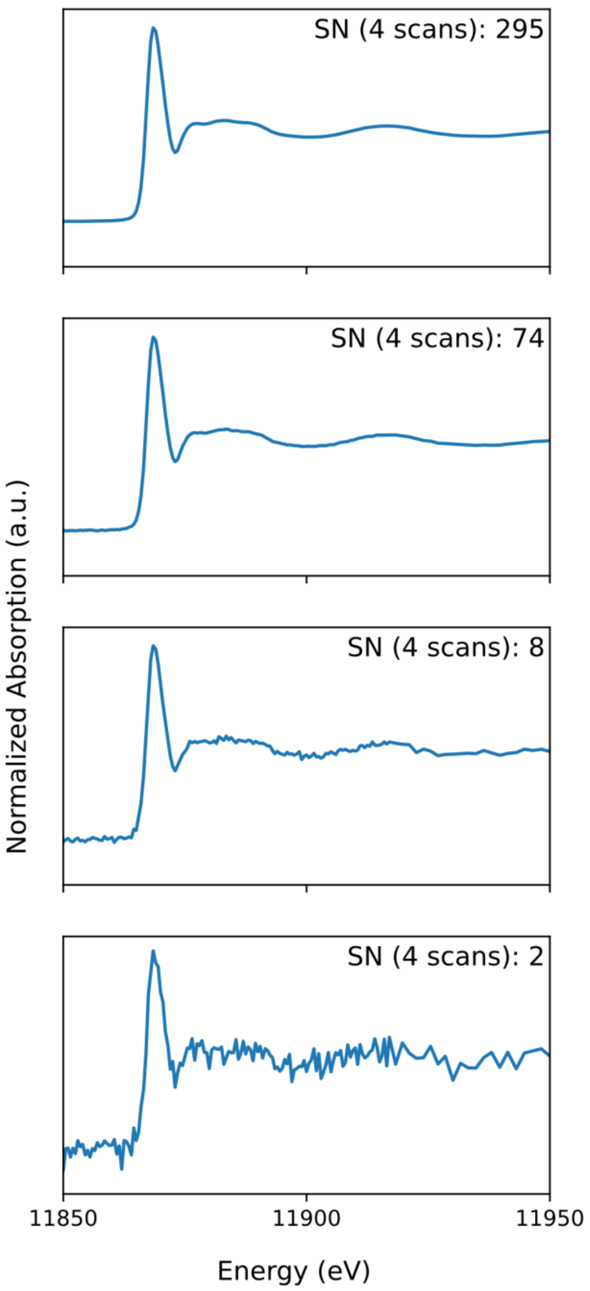

Figure S6. Top panel: Arsenopyrite reference spectra as measured in HERFD with a signal-to-noise (SN) ratio of 295 for 4 merged scans. The panels below show the arsenopyrite reference spectra with added noised to simulate SN ratios of 74, 8, and 2.

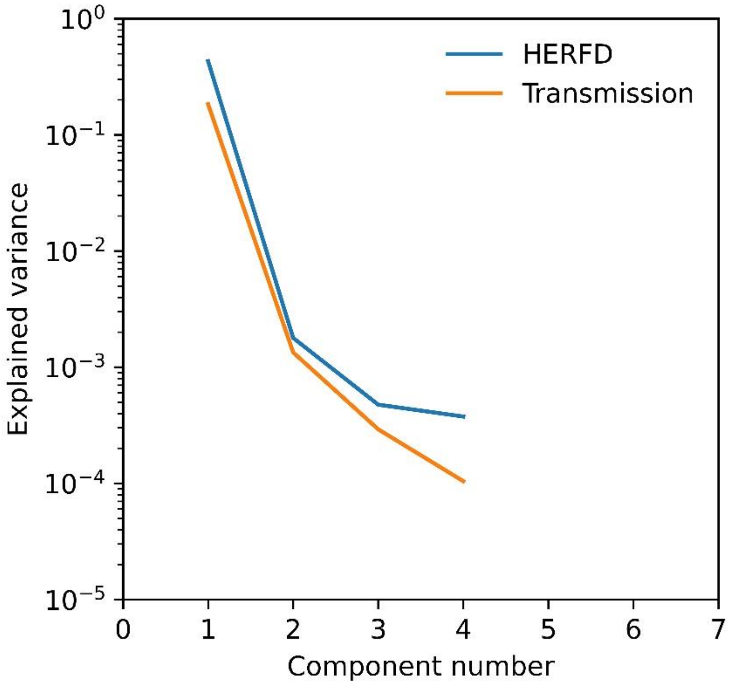

Figure S7. The scree plot for PCA components and the associated explained variance for both HERFD and transmission datasets of mine waste samples (n=7).

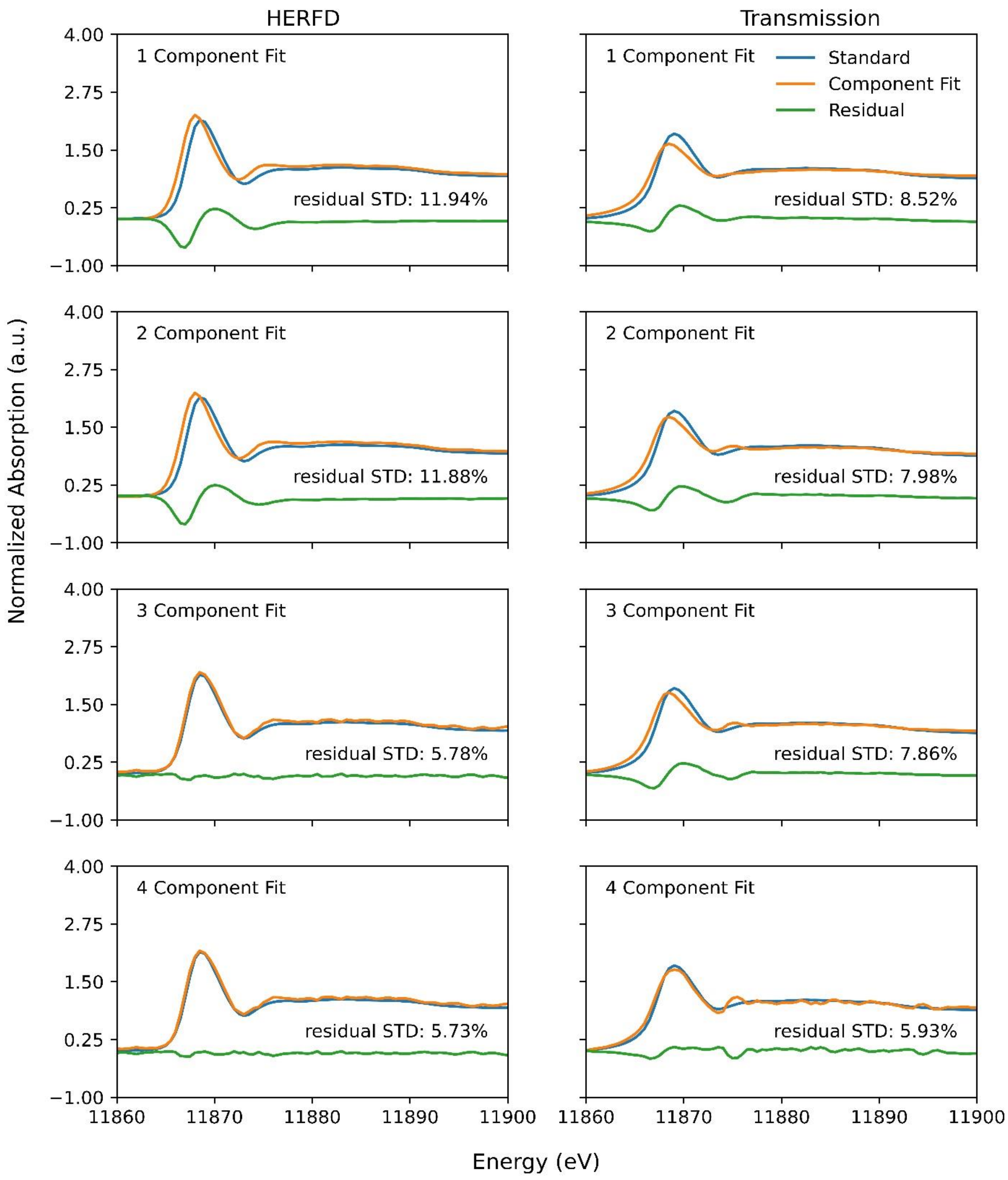

Figure S8. Demonstration of target transformations of the arsenopyrite standard, in both HERFD and transmission-detection, with principal components from the HERFD and TFY-detected mine waste sample datasets (n=7), respectively.

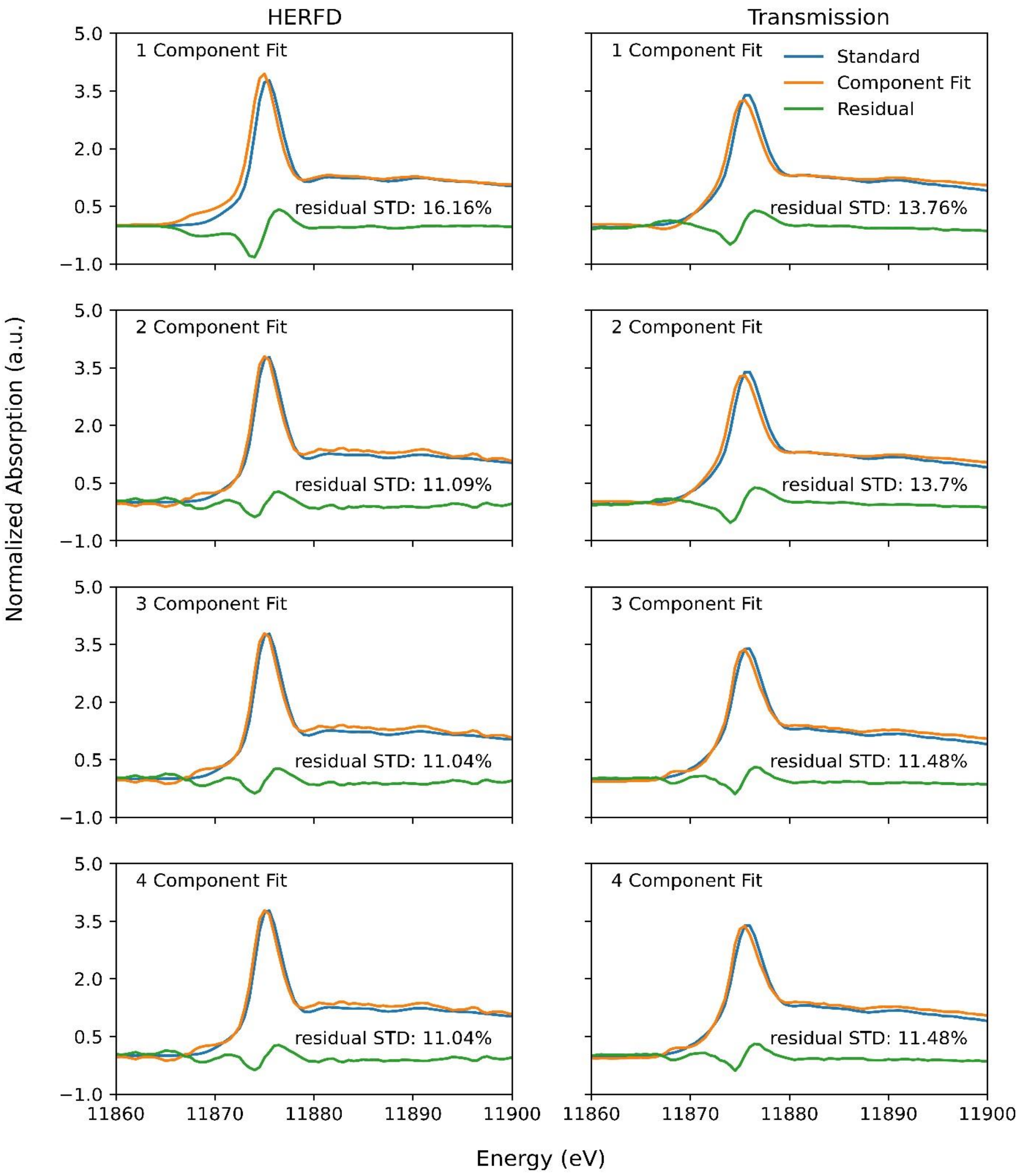

Figure S9. Demonstration of target transformations of the scorodite standard, in both HERFD and transmission-detection, with principal components from the HERFD and TFY-detected mine waste sample datasets (n=7), respectively**.**

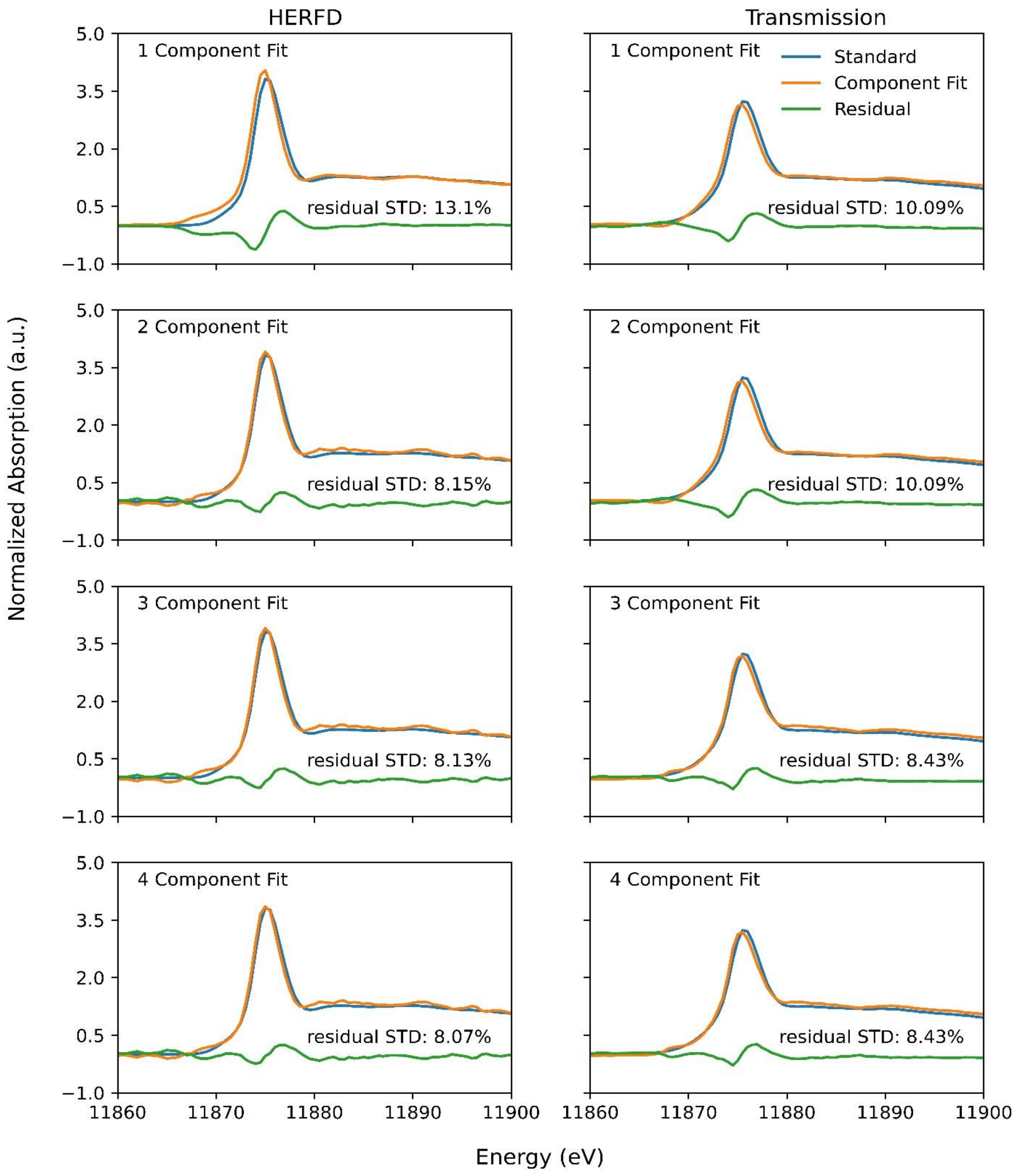

Figure S10. Demonstration of target transformations of the kankite standard, in both HERFD and transmission-detection, with principal components from the HERFD and TFY-detected mine waste sample datasets (n=7), respectively.

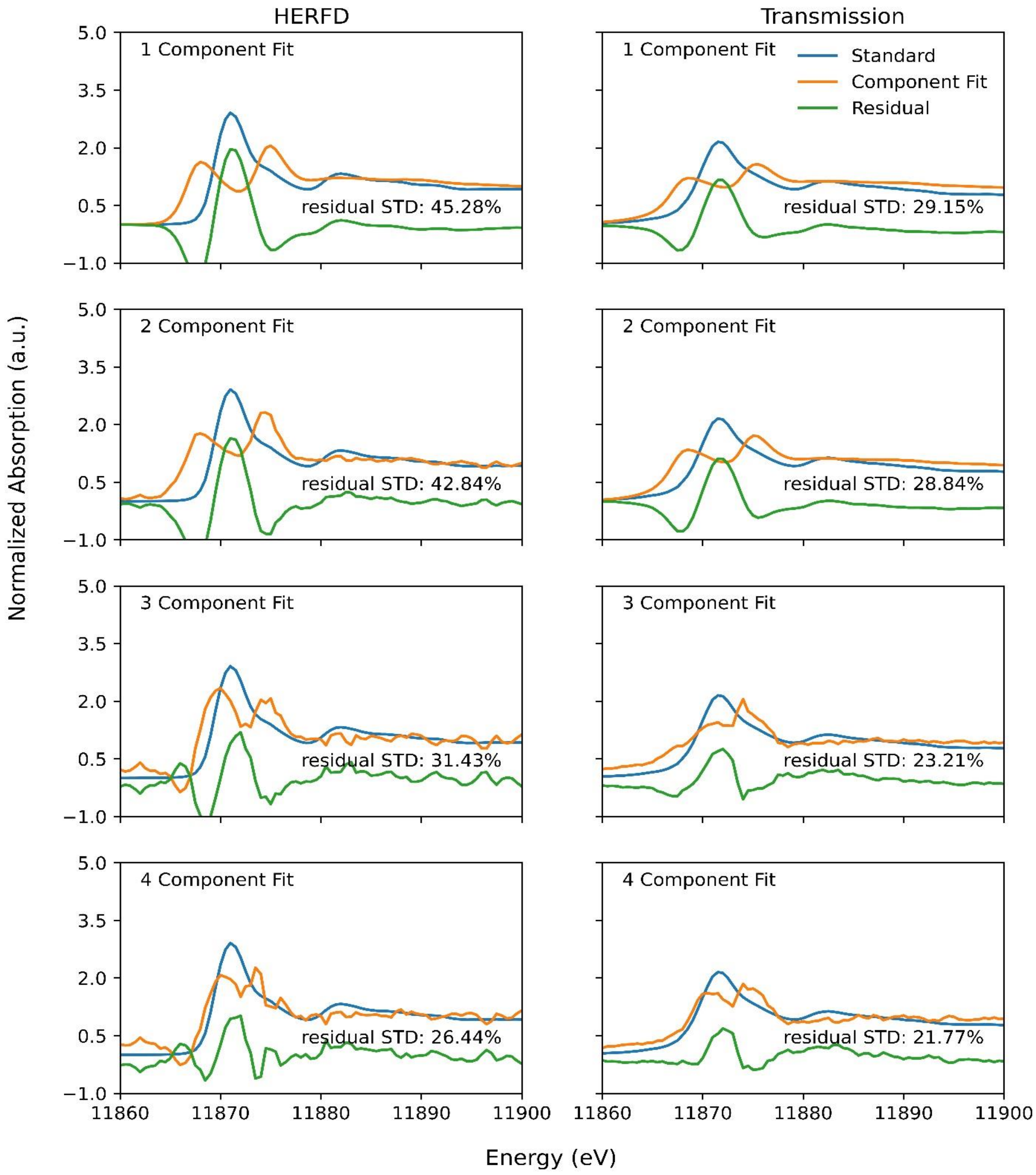

Figure S11. Demonstration of target transformations of the arsenolite standard, in both HERFD and transmission-detection, with principal components from the HERFD and TFY-detected mine waste sample datasets (n=7), respectively.

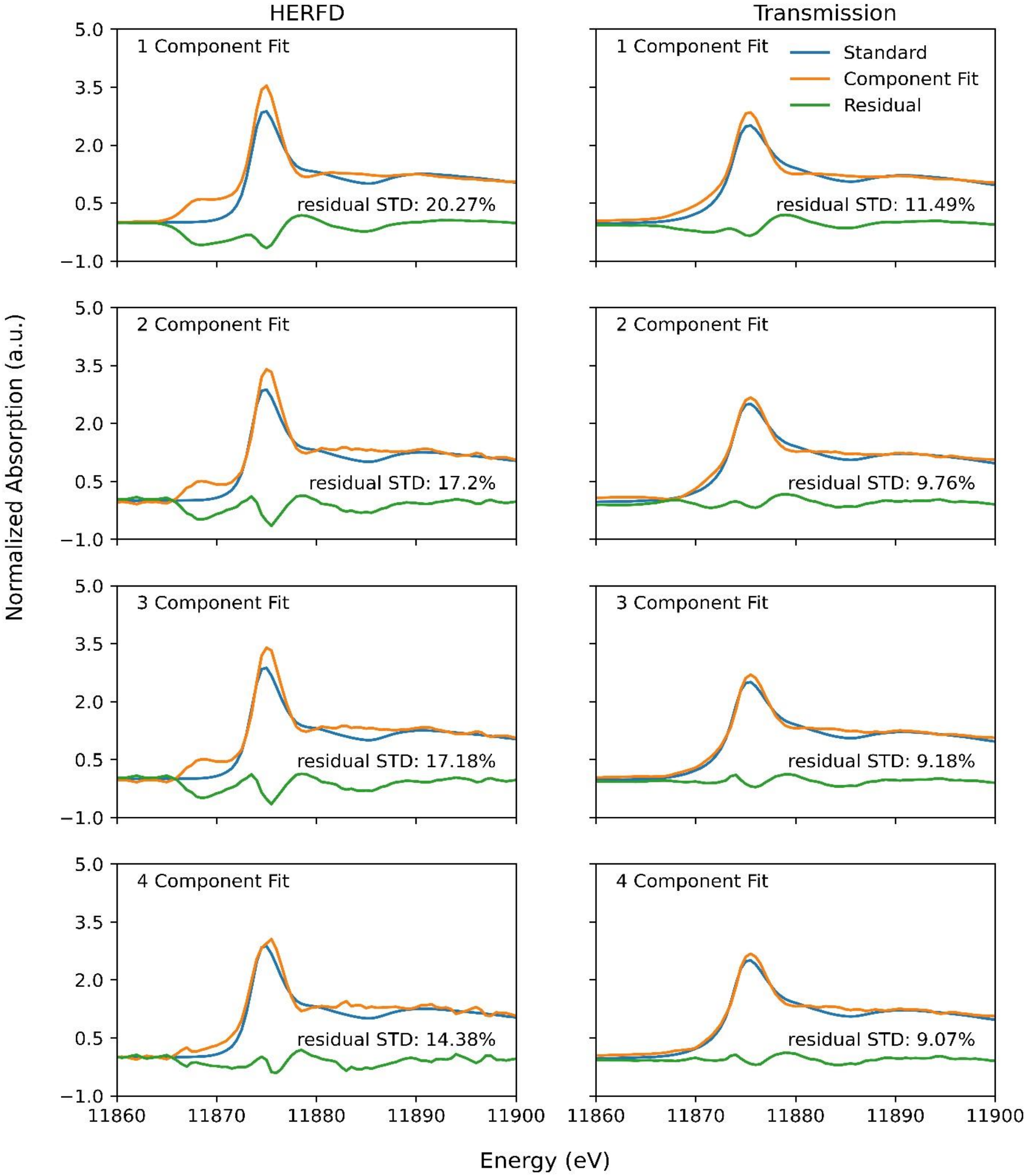

Figure S12. Demonstration of target transformations of the sodium arsenate standard, in both HERFD and transmission-detection, with principal components from the HERFD and TFY-detected mine waste sample datasets (n=7), respectively.

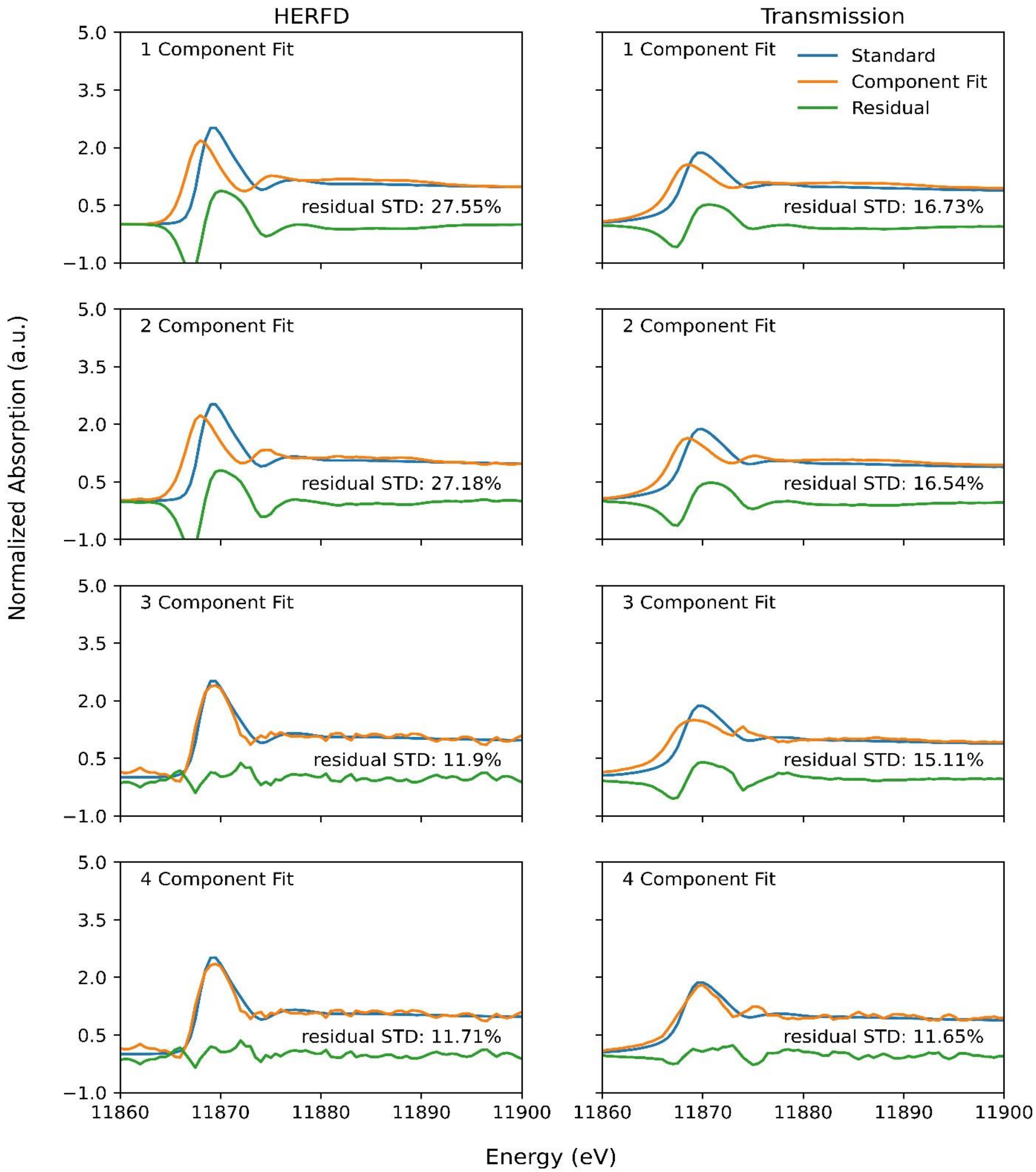

Figure S13. Demonstration of target transformations of the getchellite standard, in both HERFD and transmission-detection, with principal components from the HERFD and TFY-detected mine waste sample datasets (n=7), respectively.

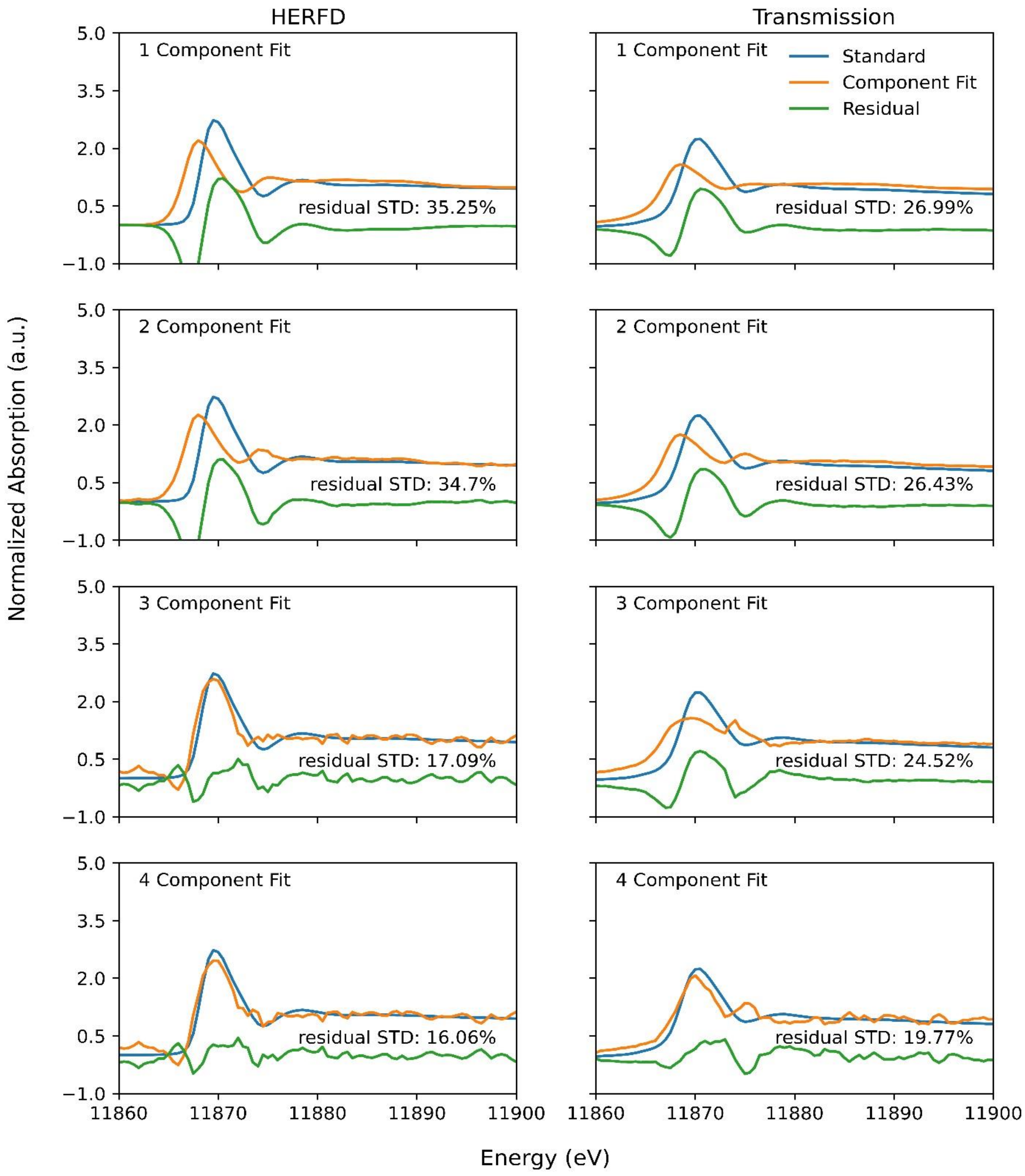

Figure S14. Demonstration of target transformations of the orpiment standard, in both HERFD and transmission-detection, with principal components from the HERFD and TFY-detected mine waste sample datasets (n=7), respectively.

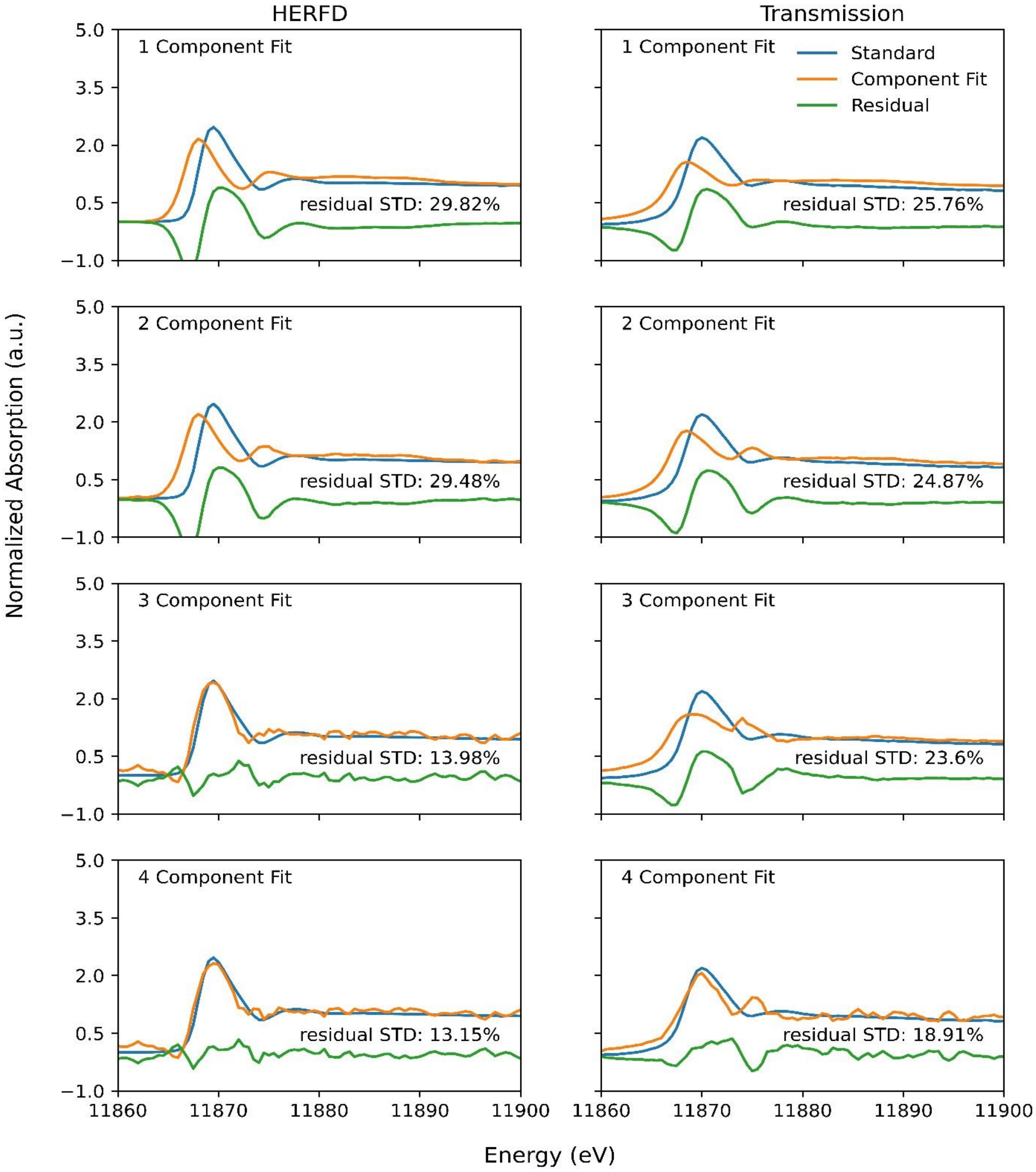

Figure S15. Demonstration of target transformations of the realgar standard, in both HERFD and transmission-detection, with principal components from the HERFD and TFY-detected mine waste sample datasets (n=7), respectively.

Table S1. SN ratios for each reference compound and sample in each detection mode. Improvement factor is the ratio of HERFD SN ratio to either transmission- or TFY-detection SN ratio.

Reference / Sample #	Signal to Noise Ratio			Improvement Factor
	HERFD	Transmission	TFY	
Arsenopyrite	295	114	-	2.6
Arsenolite	156	91	-	1.7
Getchellite	291	148	-	2.0
Kankite	79	33	-	2.4
Orpiment	232	158	-	1.5
Realgar	255	92	-	2.8
Sodium arsenate	107	27	-	4.0
Scorodite	99	25	-	4.0
1	92	-	121	0.8
2	133	-	164	0.8
3	109	-	122	0.9
4	51	-	64	0.8
5	49	-	68	0.7
6	176	-	160	1.1
7	135	-	212	0.6
8	156	-	265	0.6
9	65	-	125	0.5
10	18	-	58	0.3
11	96	-	118	0.8
12	41	-	55	0.7
13	223	-	144	1.6
14	98	-	107	0.9
15	45	-	73	0.6
16	6	-	26	0.2
17	283	-	262	1.1
18	123	-	162	0.8
19	98	-	55	1.8
20	173	-	112	1.5

21	91	-	60	1.5
22	63	-	132	0.5
23	16	-	99	0.2
24	89	-	117	0.8
25	19	-	29	0.6
26	50	-	43	1.2
27	159	-	183	0.9
28	55	-	87	0.6
29	67	-	98	0.7
30	7	-	25	0.3

Table S2. Major elemental concentrations in representative samples from Long Lake from whole rock digestion. Concentrations of Si an O were not measured with this technique. Other elements are present at lower wt%. Data from Verbuyst, 2020.

Sample Type	As (wt%)	Fe (wt%)	S (wt%)	Al (wt%)
High – As	4.37	5.12	3.42	0.15
Moderate – As	2.18	6.81	2.85	0.44
Low – As	0.10	1.76	0.09	2.37

Table S3. LCF results for all Long Lake samples analyzed, As(-I) is represented by arsenopyrite, As(III) – S is represented by getchellite, orpiment and realgar resulted in poorer quality fits, As(III) – O is represented by arsenolite, As(V) is the sum of scorodite, kankite, and sodium arsenate. R^2 is the goodness-of-fit parameter, N is the number of components in the fit.

Sample	TFY							HERFD						
	As(-I)	As(III) – S	As(III) – O	As(V)	R^2	N	Shift (eV)	As(-I)	As(III) – S	As(III) – O	As(V)	R^2	N	Shift (eV)
1	29 ± 1			71 ± 1	0.0081	2	0.50	20 ± 1			80 ± 1	0.0036	2	0.50
2	92 ± 2			8 ± 1	0.0128	2	0.30	96 ± 1			4 ± 1	0.0002	2	0.30
3	94 ± 1			6 ± 1	0.0085	2	0.50	98 ± 1			2 ± 1	0.0004	2	0.50
4	85 ± 1			15 ± 1	0.0081	2	0.50	87 ± 1			13 ± 1	0.0046	2	0.50
5	30 ± 6	31 ± 6	19 ± 6	21 ± 6	0.0040	4	0.50	33 ± 3	33 ± 4	17 ± 2	17 ± 1	0.0039	4	0.50
6	66 ± 1		22 ± 1	12 ± 1	0.0027	3	0.00	74 ± 2		16 ± 1	10 ± 1	0.0060	3	0.00
7	73 ± 2	18 ± 2		9 ± 1	0.0023	3	0.00	81 ± 2	13 ± 2		5 ± 1	0.0034	3	0.00
8	9 ± 3	44 ± 5	30 ± 2	17 ± 1	0.0028	4	0.00	10 ± 6	43 ± 7	29 ± 3	18 ± 2	0.0142	4	0.00
9	64 ± 1		19 ± 1	17 ± 1	0.0021	3	0.00	71 ± 1		14 ± 1	15 ± 1	0.0054	3	0.00
10	21 ± 1			79 ± 1	0.0031	2	0.15	13 ± 1			87 ± 1	0.0065	2	0.15
11	13 ± 3		20 ± 3	67 ± 2	0.0168	3	0.30			22 ± 1	78 ± 5	0.0029	3	0.30
12	31 ± 1		38 ± 2	31 ± 1	0.0040	3	0.00	33 ± 2		33 ± 2	34 ± 1	0.0160	3	0.00
13	86 ± 2	6 ± 3		8 ± 1	0.0024	3	0.15	91 ± 2	5 ± 2		5 ± 1	0.0025	3	0.15
14	22 ± 2	72 ± 3		7 ± 1	0.0024	3	0.00	28 ± 4	69 ± 3		3 ± 1	0.0086	3	0.00
15	9 ± 4	86 ± 4		6 ± 1	0.0064	3	0.00	20 ± 5	76 ± 5		4 ± 2	0.0184	3	0.00
16	65 ± 2		19 ± 2	16 ± 1	0.0079	3	0.00	74 ± 2		10 ± 2	16 ± 2	0.0188	3	0.00
17	88 ± 2		6 ± 2	6 ± 1	0.0070	3	0.20	95 ± 1		2 ± 1	3 ± 1	0.0004	3	0.20
18	66 ± 2		7 ± 2	27 ± 1	0.0040	3	0.00	68 ± 1		5 ± 1	27 ± 1	0.0041	3	0.00
19	9 ± 2		42 ± 2	49 ± 1	0.0036	3	0.00			44 ± 1	56 ± 1	0.0071	2	0.00
20	93 ± 1			7 ± 1	0.0056	2	0.00	98 ± 1	2 ± 1			0.0010	2	0.00
21	69 ± 2		15 ± 2	16 ± 1	0.0048	3	0.00	75 ± 2		10 ± 2	15 ± 1	0.0083	3	0.00
22	9 ± 2	68 ± 3	14 ± 1	9 ± 1	0.0006	4	0.00	10 ± 4	70 ± 5	13 ± 2	7 ± 1	0.0068	4	0.00
23	74 ± 1		8 ± 1	19 ± 1	0.0017	3	0.00	79 ± 2		3 ± 2	17 ± 1	0.0076	3	0.00
24	16 ± 2			84 ± 10	0.0120	3	0.30	5 ± 1			95 ± 2	0.0005	3	0.30
25	15 ± 2			85 ± 2	0.0125	2	0.30				100 ± 1	0.0071	1	0.30
26	93 ± 2			7 ± 1	0.0114	2	0.25	97 ± 1			3 ± 1	0.0003	2	0.25
27	85 ± 2		7 ± 2	8 ± 2	0.0072	3	0.15	95 ± 1		2 ± 1	3 ± 1	0.0005	3	0.15
28	82 ± 2		10 ± 2	7 ± 1	0.0094	3	0.40	92 ± 1		3 ± 1	4 ± 1	0.0009	3	0.40
29		92 ± 1		8 ± 1	0.0047	2	0.00		92 ± 2		8 ± 1	0.0157	2	0.00
30	78 ± 1		9 ± 1	13 ± 1	0.0008	3	0.50	88 ± 1			12 ± 1	0.0049	2	0.50
